# Enhancer-promoter interactions are reconfigured through the formation of long-range multiway hubs as mouse ES cells exit pluripotency

**DOI:** 10.1016/j.molcel.2024.02.015

**Published:** 2024-03-14

**Authors:** David Lando, Xiaoyan Ma, Yang Cao, Aleksandra Jartseva, Tim J. Stevens, Wayne Boucher, Nicola Reynolds, Bertille Montibus, Dominic Hall, Andreas Lackner, Ramy Ragheb, Martin Leeb, Brian D. Hendrich, Ernest D. Laue

**Affiliations:** 1 Department of Biochemistry, University of Cambridge, Cambridge CB2 1GA, UK; 2 MRC Laboratory of Molecular Biology, Cambridge Biomedical Campus, Cambridge CB2 0QH, UK; 3 Cambridge Stem Cell Institute, Jeffrey Cheah Biomedical Centre, Cambridge CB2 0AW, UK; 4 Max Perutz Laboratories Vienna, University of Vienna, Vienna Biocenter, Vienna, Austria

## Abstract

Enhancers bind transcription factors, chromatin regulators, and non-coding transcripts to modulate the expression of target genes. Here, we report 3D genome structures of single mouse ES cells as they are induced to exit pluripotency and transition through a formative stage prior to undergoing neuroectodermal differentiation. We find that there is a remarkable reorganization of 3D genome structure where inter-chromosomal intermingling increases dramatically in the formative state. This intermingling is associated with the formation of a large number of multiway hubs that bring together enhancers and promoters with similar chromatin states from typically 5–8 distant chromosomal sites that are often separated by many Mb from each other. In the formative state, genes important for pluripotency exit establish contacts with emerging enhancers within these multiway hubs, suggesting that the structural changes we have observed may play an important role in modulating transcription and establishing new cell identities.

## Graphical abstract

**Graphical abstract:**
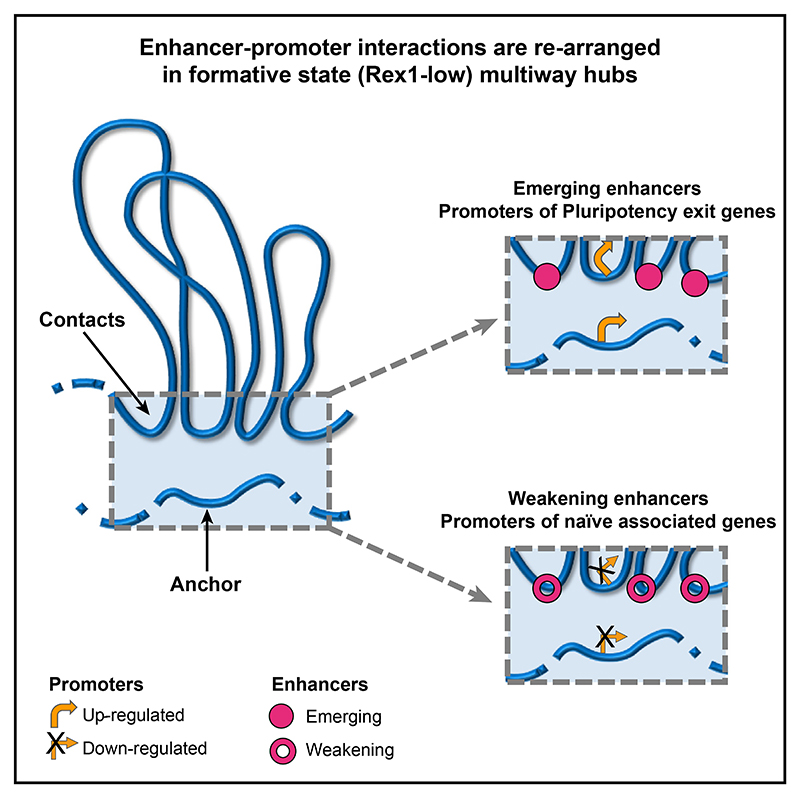

## Introduction

**Figure 1:**
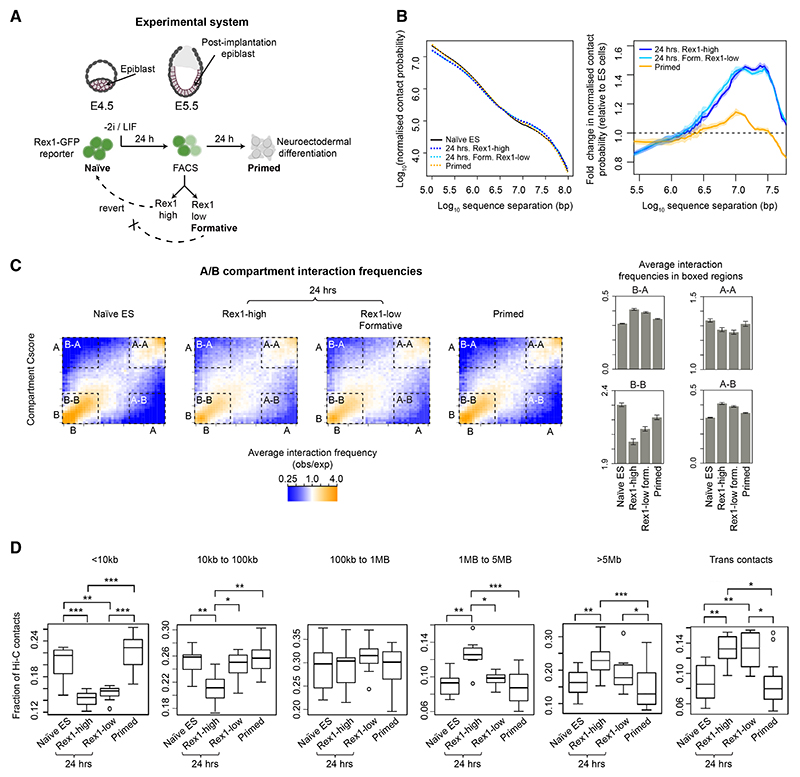
The 3D genome of mouse ES cells goes through a structural transition in the formative state (A) 24 h after two inhibitors (of the MEK/ERK pathway and glycogen synthase kinase-3) and leukemia inhibitory factor (2i/LIF) are withdrawn, the Rex1 (*Zfp42*) gene becomes silenced as cells exit naive pluripotency: at this time, a proportion of cells have progressed into the formative (Rex1-low) state, whereas the remainder (Rex1-high cells) are still exiting naive pluripotency.^4^ A further 24 h later, as *Zfp42* expression is lost in all cells, mouse ES cells enter a primed state that in basal media prepares them to differentiate down the neuroectodermal lineage. (B) (Left) Plots of log_10_ contact probability against log_10_ intra-chromosomal sequence separation determined using in-nucleus Hi-C experiments carried out on large numbers of cells. (Right) Expansion showing the fold change in Hi-C contact probability relative to that in naive ES cells. Error bands depict the 95% confidence interval. (C) Saddle plots showing the normalized average A/B compartment interaction frequencies between pairs of 25 kb bins, arranged by their A or B compartment Cscores (see STAR Methods for the definition of the Cscore). The bar charts show the average normalized Cscores from within the boxed A/B compartment regions. Bootstrap estimates of the standard deviation were obtained from 200 random samples of 20% of the data. (D) Boxplots showing the fraction of contacts in the single nuclei Hi-C datasets that are <10 kb apart in the same chromosome (left); between 10 and 100 kb, 100 kb and 1 Mb, 1 and 5 Mb, or >5 Mb apart in the same chromosome (middle); or between different chromosomes (right). FDR-adjusted Mann Whitney U test p values: * p < 0.05; ** p < 0.01; *** p < 0.001. Related to Figure S1.

Embryonic stem (ES) cells can differentiate into all the different lineages of a mature organism. Naive and primed ES cells *in vitro* correspond to the pre- and post-implantation populations in the embryo,^1,2^ and they are developmentally linked to each other via progression through a “formative” transition state where cells mature in response to inductive cues (Figure 1A).^3^ In this formative state, there is a rewiring of transcription factor networks—this results in the downregulation of genes required for naive pluripotency, such as *Nanog* and *Klf4*, and the upregulation of genes associated with the primed epiblast, such as *Otx2* and those encoding the *de novo* methyltransferases *Dnmt3a/b*. After transition through the formative state, epiblast cells can be induced to form primordial germ cells, and subsequently, they become progressively specified toward different cell fates.^4,5–7^ Stimulated by the observation that nuclei of formative state cells have different material properties to those of either naive or primed ES cells,^8^ we have explored how genome structure changes in this crucial transition state.

In recent years, a large number of experiments employing light microscopy and either biochemical ligation-based (chromatin condensation capture [3C]) or ligation-independent methods (split-pool recognition of interactions by tag extension [SPRITE] or genome architecture mapping [GAM]) have suggested that three key mechanisms—compartmentalization, loop extrusion, and tethering—play a critical role in organizing 3D genome structure in the nucleus. Biochemical experiments carried out on large numbers of cells have illuminated important features of chromosome and genome structure that are conserved in many (or all) cells. In particular, they have shown that the genome is segregated into the following: (1) A and B compartments (eu- and hetero-chromatin); (2) megabase-scale topologically associating domains (TADs), which have a higher frequency of intra-compared to inter-domain chromatin interactions; and (3) loops where specific regions of the genome contact each other more frequently. Both TADs and loops are generated via loop extrusion mechanisms leading to convergent CTCF sites becoming linked through Cohesin-mediated bridging. Importantly, enhancers can be brought into physical proximity with their target promoter by this process. Alongside these studies, a combination of imaging- and sequencing-based methods have shown that Lamin-associated domains are either tethered to the nuclear periphery or sequestered around the nucleolus. For recent reviews of the biochemical techniques used to study genome organization, the mechanisms of chromosome folding and nuclear organization that have been uncovered, and the mechanisms of enhancer action, see Kempfer and Pombo,^9^ Mirny and Dekker,^10^ and Panigrahi and O’Malley,^11^ respectively.

The extraordinary variability of chromosome/genome folding from cell to cell means that single-cell versions of 3C experiments are needed for studies of 3D structure.^12–18^ Many structural features, such as the intermingling between chromosomes and the interactions between individual enhancers and genes, are hidden in biochemical experiments carried out on populations of cells because they measure a statistical average of contact probability across many (often millions) of cells. By contrast, single-cell experiments allow one to unambiguously map individual pairwise contacts and therefore study enhancer-promoter interactions in a particular cell. In addition, contact data from a single-cell Hi-C experiment (one of the 3C methods) can be used as constraints to calculate 3D structures of how chromosomes/intact genomes fold.^12–14,17^ 3D structure determination is also useful because it allows one to distinguish real long-range contacts from noise in single-cell Hi-C experiments. Moreover, it is possible to determine the relative 3D positions of genes that are far away from each other in the nucleus and that do not contact each other.^19^

The variability of chromosome and genome structure from cell to cell that has been observed in single-cell Hi-C experiments has been confirmed by recent advances combining highly multiplexed fluorescence *in situ* hybridization (FISH) and super-resolution imaging, which allows the tracing of chromatin structure in single cells. Either individual enhancer-promoter interactions can be imaged at high resolution or the organization of, e.g., TADs within a chromosome can be studied at a lower resolution. In further developments of this technology, chromatin tracing has been combined with studies of RNA levels of hundreds of genes. For a review of the large number of exciting new methods that have recently been developed to image the 3D genome and trace chromatin structure, see Hu and Wang.^20^

Previous studies have shown that there are significant changes in chromosome structure during the parental to embryo switch as gene expression is first activated.^15,21,22^ In addition, changes in chromosome structure that correlate with changes in transcription can be observed over days and weeks in longer-term development.^23–25^ However, it has been unclear how enhancer-promoter interactions change genome wide as cells undergo rapid alterations in the expression of large numbers of genes during cell fate transitions (over 1–2 cell cycles). In this work, we wanted to study how 3D genome structure changes as cells exit pluripotency and to carry out an unbiased genome-wide study of enhancer-promoter interactions in single mouse ES cells in the naive, formative, and primed states of pluripotency. Unexpectedly, we find that there is a major reorganization of 3D genome structure as cells transition from the naive into the formative pluripotent state. We find that promoters of genes whose expression levels are changing during this transition become co-located with emerging enhancers that reside in sequences that are often many megabases distant in structures we call “multiway hubs.” These multiway hubs are located at the interface between the parts of the chromosome that are buried and regions that become highly intermingled with other chromosomes. They are dependent upon the DNA methylation machinery for their formation, and they correlate with areas of Polycomb repressive complex activity. By determining 3D genome structures in single cells, we provide evidence that promoters of genes required for pluripotency exit establish unexpectedly long-range interactions with different emerging enhancers in different individual cells to (we suggest) drive the transcriptional changes necessary for lineage commitment.

## Results

### Mouse ES cells undergo a structural transition shortly after the onset of differentiation

We exploited a culture-based system that recapitulates the early *in vivo* stages of mouse differentiation down the neuroectodermal lineage^4,7^ to ask whether genome structure changes as cells exit from naive pluripotency into the formative state (Figure 1A). In-nucleus Hi-C experiments (Table S1) using mouse ES cells expressing a Rex1-destablized GFP fusion^26^ revealed that 24 h after induction of differentiation, the genome of mouse ES cells goes through a distinct structural transition in the Rex1 (*Zfp42*)-high (less differentiated) and Rex1-low (more differentiated, formative) states, with a decrease in short- and an increase in long-range intra-chromosomal Hi-C contacts (<1 and >5 Mb, respectively, Figure 1B). 24 h later, Hi-C contact probability reverts to naive ES-like levels as cells progress toward the primed state (Figure 1B). We also found that formation of the 24 h transition state involves a significant increase in A/B compartment mixing (increased A-B and B-A interactions, false discovery rate [FDR] Wilcoxon rank-sum tests p < 10^−10^, Figure 1C)—i.e., in interactions between euchromatin and heterochromatin.^10^

**Figure 2:**
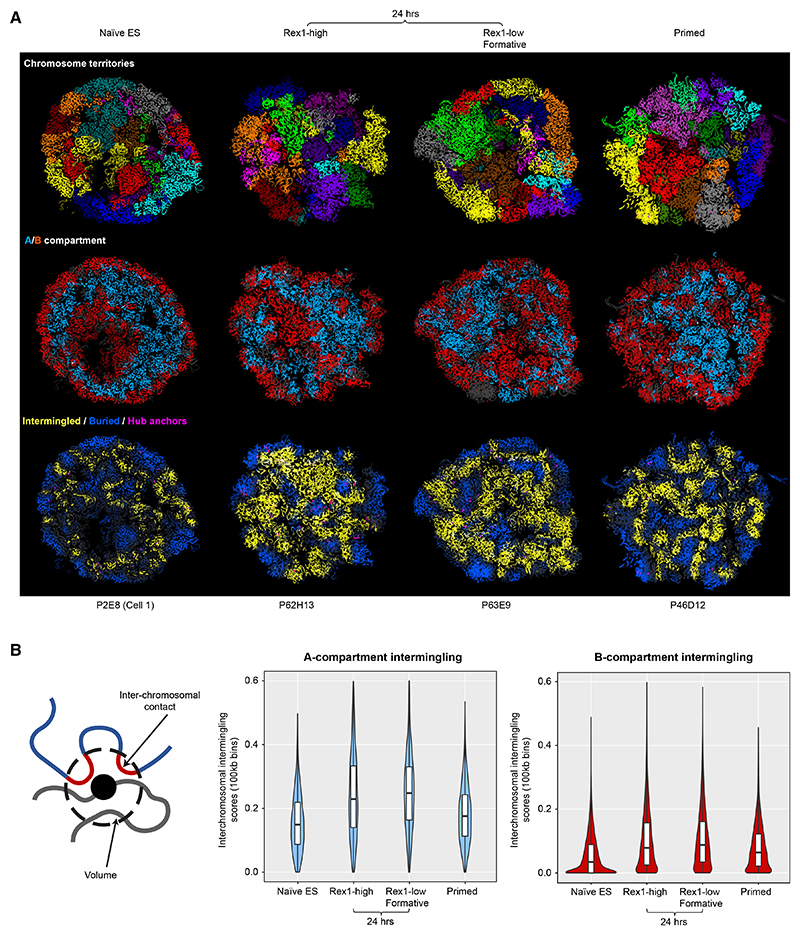
The structural transition in the formative state involves increased chromosomal intermingling in the A compartment (A) Slices through 3D genome structures of representative single G1 phase nuclei, where the individual chromosomes are colored differently (top), colored blue or red depending on whether a particular region of a chromosome is in the A or B compartment, respectively (middle), or colored yellow or blue depending on whether that particular region of the chromosome is either intermingled with another chromosome or buried (bottom). Regions transitioning between the two are colored gray. Five independently calculated 25 kb structures are superimposed to show that the structures are well determined by the Hi-C data. (B) (Left) An intermingling score was defined as the fraction of beads (in the polymer model of the 3D genome structure) that are located on different chromosomes and in close spatial proximity. (Right) Violin plots of the inter-chromosome intermingling scores of genomic loci determined from the 3D structures show a significant increase in intermingling of A (and to a lesser extent B) compartment regions in 24 h state cells. Related to Figure S2.

To understand these structural changes, we carried out single nucleus (sn) Hi-C experiments to determine 3D genome structures^12,14^ (Table S2). In both Rex1-high and -low cells, we observed an increase in inter-chromosomal contacts in the 24 h state as well as a loss of short-range and an increase in long-range (>5 Mb) intra-chromosomal interactions (Figure 1D). Calculation of 3D genome structures of individual G1 phase nuclei (Table S3) showed that both naive ES and primed cells have a characteristic Rabl configuration of chromosomes, but this is very disrupted in the 24 h state (Figure S1). Mapping of the A/B compartments onto the 3D single-cell structures showed that the naive ES cell structure—which comprises a ring of B compartment at the nuclear periphery and around the nucleolus sandwiching the A compartment in the interior^14^—becomes fragmented in the 24 h state (Figure 2A). In primed cells, A/B compartment structure partially reverts toward that seen in naive ES cells. Strikingly, we found that the structural disruption in the 24 h state involves a significant increase in inter-chromosomal intermingling in the A-compartment (~63% in Rex1-high and ~81% in Rex1-low [formative] cells, Mann Whitney U test for both cases p < 10^−15^) compared with naive ES cells (Figures 2A, 2B, and S2; see also Videos S1, S2, S3, and S4). This decompaction of chromatin structure in the 24 h state is supported by fluorescence lifetime imaging studies,^27^ and the shorter cell cycle in formative state cells^28^ suggested that this greater intermingling does not result from chromosomes’ having more time to unfold in G1 following mitosis.

**Figure 3:**
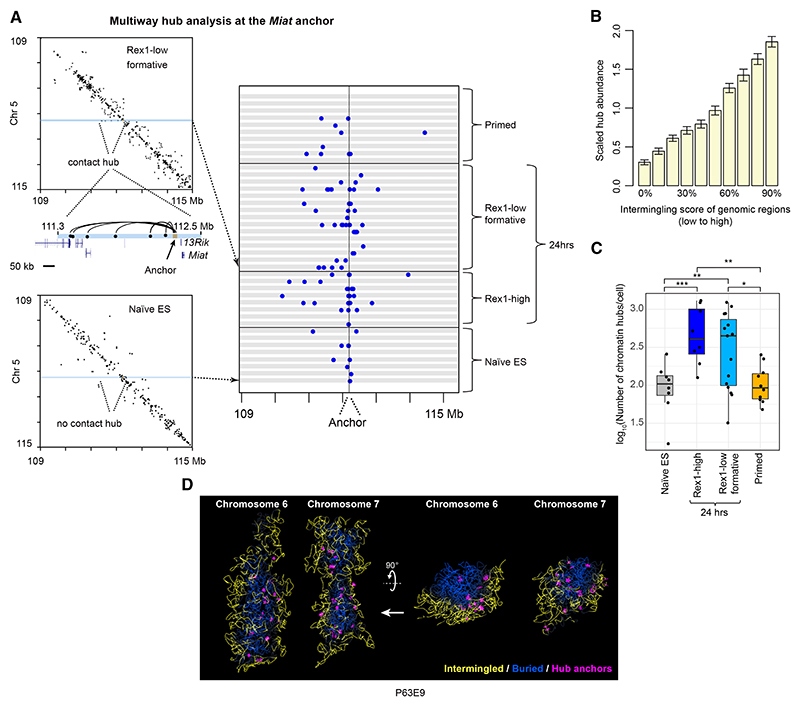
Multiple Hi-C contacts to anchor regions in 3D genome structures of single cells identify multiway hubs (A) (Left) An example of a multiway chromatin hub whose anchor is nearby the *Miat* ncRNA gene. (Right) Contacts made from the anchor in different cells and stages of differentiation—each horizontal gray box shows the same small section of the contact map (see left). (B) After dividing the genome into 10 equal-sized groups according to the level of chromosome intermingling, the abundance of hubs was found for each group and scaled by the average number of hubs across all the groups. Bootstrap estimates of the standard deviation of hub abundance within each intermingling group were obtained from 2,000 random samples of 30% of the data. (C) Boxplots showing the number of multiway hubs identified in individual single cells at different stages of differentiation. FDR-adjusted Mann Whitney U test p values: * p < 0.05; ** p < 0.01; *** p < 0.001. (D) Exploded views of chromosomes 6 and 7 in two different orientations (left and right) from the 3D structure of the Rex1-low formative state cell (P63E9) in Figure 2A. The chromosomes are colored as in Figure 2A, and the positions of the anchors of the multiway hubs are indicated by magenta stars. A single 100 kb structure is shown so that the positions of the hub anchors can be seen more clearly. Related to Figures S3 and S4.

Previous work carried out on populations of cells has shown that “stripes” of Hi-C contacts arise from the extrusion of DNA loops by Cohesin.^29–31^ Hi-C and higher resolution Micro-C data additionally revealed the presence of “dots” within such “stripes.”^32–34^ In our snHi-C experiments, we were often able to directly observe a series of Hi-C contacts aligned along the same horizontal or vertical axis in the contact maps (see Figures 3A and S3). In snHi-C, these series of contacts all originate from the same single haploid nucleus, and they correspond to “multiway hubs” where genomic interactions form between an “anchor” (a single small ~40 kb sequence) and multiple (typically 5–8) “contact” sites. Multiway hubs lead to the formation of a compact chromatin domain within the structure, and they cannot be detected in population experiments that identify individual loops in different cells. Generation of 3D structures from snHi-C data additionally allows us to study the spatial arrangement of these hubs that are often far apart from each other in the nucleus.

Despite the enormous variation in 3D genome structure from cell to cell, we found that some loci form multiway hubs in multiple cells (including the examples in Figures 3A and S3). However, a particular anchor locus makes different contacts in different cells, suggesting that the 3D structures provide snap-shots of dynamic processes that lead to the generation of these multiway hubs. Further analysis showed that inter-chromosomal intermingling and the formation of multiway hubs are significantly associated with each other (Figure 3B). As with inter-chromosomal intermingling, in both Rex1-high and -low cells, there is a very significant increase in the abundance of multiway hubs in 24 h cells compared with either the naive or primed states (Figure 3C). Strikingly, inspection of the 3D structures showed that multiway chromatin hubs are found at the interface between the region of the chromosome that is buried (mainly B compartment) and the regions that are intermingled with other chromosomes (mainly A compartment) (Figure 3D). This observation was quantified by showing that hubs tend to be located in the regions of chromosomes where the intermingling density is intermediate between that of the A compartment (most intermingled) and B compartment (most buried) (Figure S4).

Although the multiway hubs that are formed and the chromosomes that intermingle with each other vary from cell to cell, the regions of a chromosome that intermingle are much more consistent—with the average intermingling scores from as few as three cells clearly matching the population average (Figure S5A). Multiway hubs fall into two groups whose maximum sequence span of intra-chromosomal contacts is either less than ~1 Mb or many Mb in length (Figure S5B). The correlation of anchor CTCF-binding site orientation with the shorter-, but not the longer-range, contacts in multiway hubs (Figure S5C) leads us to hypothesize that although the shorter-range contacts likely form through CTCF/Cohesin-mediated loop extrusion,^29,35,36^ the much longer range contacts may be generated through interactions between genomic regions bound by either transcription factors or Polycomb (PRC1/2) complexes.^37–39^

Thus, 3D genome structure undergoes a significant transition in the 24 h state (in both Rex1-high and -low cells), with a very substantial increase in inter-chromosomal intermingling and the formation of multiway hubs, which are typically found at the boundary between the buried and intermingled regions of a chromosome.

### Regions of inter-chromosomal intermingling and multiway hubs are enriched in markers of active gene expression and Polycomb-mediated heterochromatin

**Figure 4:**
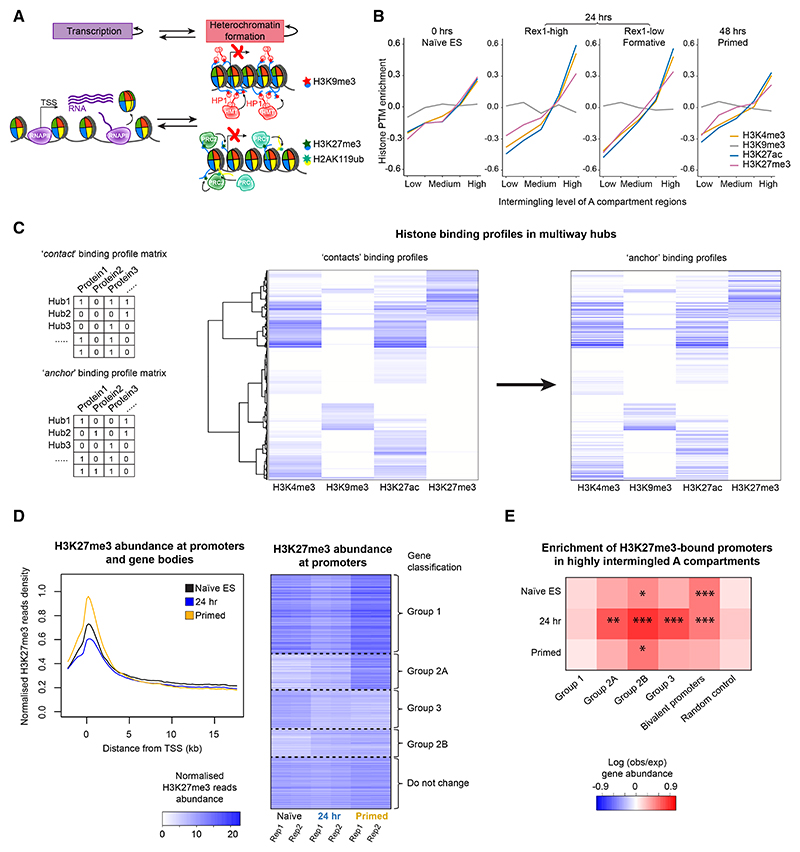
Anchors and contacts in multiway hubs bind similar post-translationally modified nucleosomes (A) Transcription and heterochromatin formation are self-reinforcing processes leading to either the deposition/modification of nucleosomes with active (H3K4me3 and H3K27ac) or repressive (H3K9me3/H3K27me3) histone marks and heritably expressed or silenced states. (B) H3K4me3, H3K27ac, and H3K27me3, but not H3K9me3, modified nucleosomes are found in highly intermingled regions of the A compartment in formative state cells. In this analysis, the A compartment of the genome was divided into 5 groups according to the intermingling level. The relative abundance of each type of histone modification was then compared to the average levels, and a Fisher’s exact test was calculated when comparing the density in the highest and the lowest intermingled regions (p < 10^−15^ for all the modifications except H3K9me3). (C) (Left) Schematic illustrating the approach used to analyze anchor or “average contact” histone protein binding profiles in individual hubs in single cells (NB—in practice, the actual number of ChIP-seq reads was used as opposed to a binary [0, 1] distinction of histone binding). (Middle) An average protein binding profile matrix was calculated for all the contacts in a hub, which were then ordered using hierarchal clustering. (Right) By keeping the row order the same, each line continues to represent the same chromatin hub but now shows the histone protein binding profile at the anchor in that hub. (D) (Left) Pileup analysis of histone H3K27me3 binding to genes, plotted in 200 bp bins from 2 kb upstream of the transcription start site (TSS) across the gene body. (Right) When looking at promoters bound by nucleosomes with the H3K27me3 modification, genes can be hierarchically clustered into four different groups depending on how their H3K27me3 binding levels change during the time course. (E) The enrichment of H3K27me3-bound promoters within genomic regions having high levels of A compartment inter-chromosomal intermingling. The colors shown indicate the log fold change in gene abundance—we compared the top 30% most intermingled A compartment regions with the bottom 30% (see STAR Methods). FDR-adjusted empirical p values: * p < 0.05; ** p < 0.01; *** p < 0.001. Related to Figures S5 and S6.

Transcription and heterochromatin formation are opposing processes whose balance at a particular gene results in the deposition or modification of nucleosomes with either active (H3K4me3 and H3K27ac) or repressive (H3K9me3 and H3K27me3) histone marks and heritable states of either active gene expression or silencing, respectively (Figure 4A). To identify different chromatin states, we carried out chromatin immunoprecipitation sequencing (ChIP-seq) experiments in naive ES, 24 h, and primed cells to study the binding of post-translationally modified histones to identify different chromatin states. We then mapped the resulting data onto the sn 3D genome structures and found that marks of active enhancers and promoters (H3K27ac and H3K4me3) and Polycomb-mediated heterochromatin (H3K27me3), but not HP1-mediated heterochromatin (H3K9me3), were all highly associated with inter-chromosomal intermingling of A compartment regions in the 24 h state, but this was much less noticeable in naive or primed cells (Figure 4B).

Hierarchical clustering of histone binding profiles in individual multiway hubs in single cells showed that “anchors” and “contacts” with very similar chromatin states cluster together in the same hub. (Cut&Run experiments [carried out without cross-linking] for two of the histone modifications [H3K4me3 and H3K27me3] confirmed this result [data not shown.]) We found that some hubs bore marks of active transcription (H3K4me3 and H3K27ac), some had marks of Polycomb-mediated hetero-chromatin (H3K27me3), and some had marks of both (Figure 4C). The smaller number of hubs bearing the H3K9me3 mark were not enriched above the background. In addition, the association of H3K27me3, but not H3K9me3, with inter-chromosomal intermingling in the 24 h state (Figure 4B) prompted us to focus on changes in the formation of PRC1/2-mediated heterochromatin.

We found that in the 24 h state, there is a global reduction in H3K27me3 levels—this decreases throughout the promoter and gene body and then builds up again at the promoter in primed cells (Figure 4D), suggesting a loss of PRC1/2-mediated hetero-chromatin and its subsequent reestablishment.^40^ We classified genes according to how H3K27 methylation levels change at their promoters and identified four major groups, whose gene ontology (GO) terms were all found to be significantly related to embryonic development, cell differentiation, and cell-cell signaling. Interestingly, the top hits for group 1 genes, which follow the global changes in H3K27me3 levels (see Figure 4D), all relate to neuroectoderm development—including the regulation of neural precursor cell proliferation, sensory system development, and synapse organization (Figure S6A). In group 2 genes, promoter H3K27me3 levels increase throughout the time course despite the global decrease in the 24 h state (Figure 4D). This group can be split into two: group 2A consists of genes whose promoter H3K27me3 levels increase in both the 24 h and primed states, whereas in group 2B the levels increase more modestly in the 24 h state (than in group 2A) and then remain stable in the primed state. We found that GO terms for group 2 genes do not relate to neuroectoderm development but are instead biased toward the meso/endodermal lineages—including embryonic organ/skeletal system development (group 2A) and tissue remodeling (group 2B) (Figure S6A). Finally, GO terms for group 3 genes are significantly related to the organization of intermediate filament-based processes and fiber organization—processes that could be of importance in early cell differentiation. It is also worth noting that GO terms for groups 1 and 2A genes relate to distinct signaling pathways (mitogen-activated protein kinase [MAPK] and BMP, respectively) (Figure S6A) (a complete list of the genes in the different H3K27me3 groups can be found in Table S4, and the GO terms relating to these different groups of genes can be found in Table S5).

Both group 2 and group 3 promoters—which, in 24 h cells, have increasing and decreasing levels of H3K27me3, respectively—are enriched in regions of high inter-chromosomal intermingling (Figure 4E). We identified bivalent promoters (marked by both H3K4me3 and H3K27me3) and tracked their formation from, and resolution into, monovalent promoters (either H3K4 or H3K27 methylated) during the time course (Figure S6B). Bivalent promoters were also found to be particularly enriched in regions of high inter-chromosomal intermingling in naive ES and 24 h cells (Figure 4E). Furthermore, promoters that are bivalent at all three stages in the time course were found to be enriched in group 1 genes, whereas those that form in the 24 h state and are maintained in the primed state are enriched in group 2B genes (Figure S6C). Finally, bivalent promoters that form only in naive ES or primed cells are enriched in either group 3 or group 2A genes, respectively (Figure S6C).

Thus, the 24 h state is characterized by an alteration in H3K27me3 levels at promoters (Ps) that are found in regions of high inter-chromosomal intermingling and multiway hubs, suggesting a switch in the balance between transcription and the formation of PRC1/2-mediated heterochromatin in these regions. Moreover, genes whose H3K27me3 levels change in different ways are associated with alternative developmental lineages—suggesting that changes in gene expression and/or PRC1/2-mediated heterochromatin formation might prime them for differential expression later in differentiation.

### Regions of inter-chromosomal intermingling and multiway hubs are enriched in genes that are required for the exit from naive pluripotency

**Figure 5:**
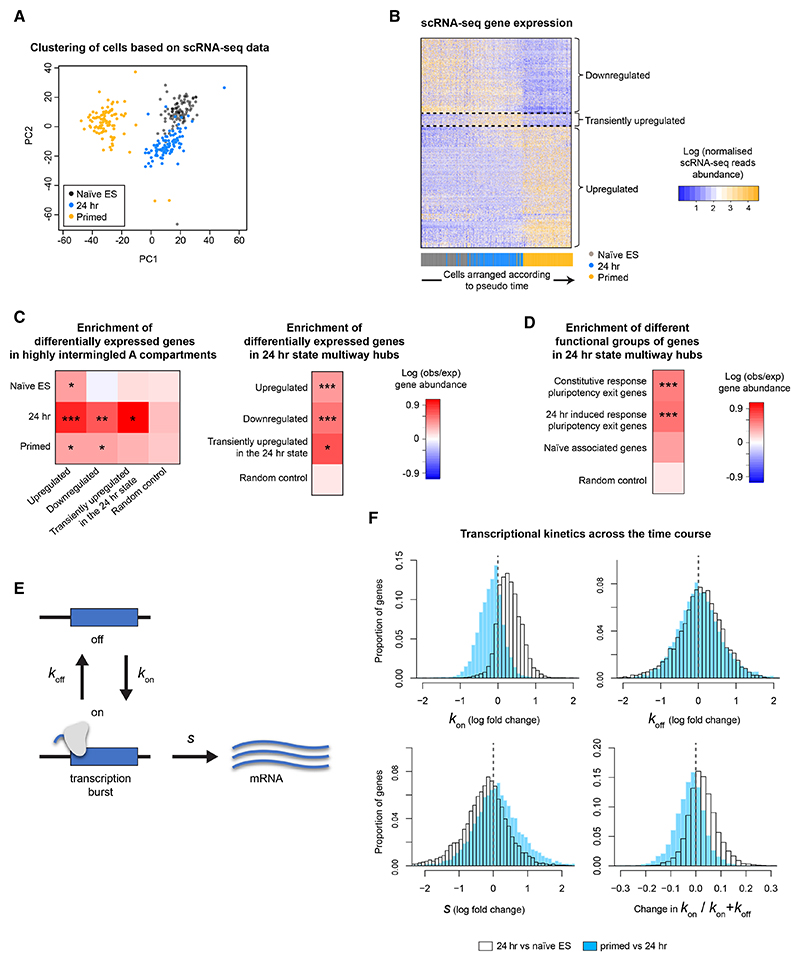
There is a global increase in transcriptional bursting in 24 h state cells (A) After principal-component analysis (PCA) analysis, single-cell (sc) RNA-seq data (0, 24, and 48 h after induction of differentiation) can be separated using k-means clustering. (B) Expression profile for differentially expressed genes in the scRNA-seq data (each cell is represented by a different column). The cells were ordered according to pseudo time (see STAR Methods), with the naive ES, 24 h, and primed cell datasets being colored black, blue, and yellow, respectively, in the bar chart. Differentially expressed genes (individual rows) were hierarchically clustered to identify three categories of genes whose expression levels change during the time course. (C) The enrichment of differentially expressed genes at different time points within either genomic regions having high levels of A compartment inter-chromosomal intermingling (left) or multiway hubs (right). (D) The enrichment of pluripotency exit and naive-associated genes in 24 h multiway hubs (very similar results were obtained when analyzing the separate Rex1-high and Rex1-low [formative state] cells [data not shown]). The colors shown in the heatmaps in (C) and (D) indicate the log fold change in gene abundance—we compared the top 30% most intermingled A compartment regions with the bottom 30% and contacts observed within 24 h state multiway hubs to the genome-wide distribution (see STAR Methods). FDR-adjusted empirical p values: * p < 0.05; ** p < 0.01; *** p < 0.001. (E) The model used to estimate kinetic parameters from the scRNA-seq data (see Kim and Marioni^41^). 1/*k*_off_ and 1/*k*_on_ describe the average time a gene spends in the active and inactive states, respectively, while *s* is the transcription rate. (F) Histograms showing the log fold changes in *k*_on_, *k*_off_, and *s* as well as the changes in the average fraction of time that genes spend in the active state (*k*_on_/(*k*_on_ + *k*_off_)) during the time course. (Wilcoxon paired tests p < 10^−15^ for all *k*_on_, *s*, and *k*_on_/(*k*_on_ + *k*_off_) comparisons of naive ES vs. 24 h and 24 h vs. primed cells. The values for *k*_off_ were p < 3.9 × 10^−12^ when comparing naive ES and 24 h cells and 5.8 × 10^−5^ when comparing 24 h and primed cells.) Related to Figures S6 and S7.

We next carried out single-cell RNA sequencing (scRNA-seq) experiments in naive ES, 24 h, and primed cells (Figures 5A and 5B). There is a good correlation between the classification of groups of genes based on changing levels of H3K27 methylation and the levels of expression in the scRNA-seq data with group 2A and group 3 genes, which respectively have increasing and decreasing levels of H3K27me3 at their promoters, being significantly enriched among genes that are down- and up-regulated during the time course (Figure S6D) (a complete list of the genes in the different scRNA-seq groups can be found in Table S6). A small proportion of genes (1.8%) whose expression levels increase in the 24 h state move from the B to the A compartment upon differentiation, but most (74.4%) are constitutive A compartment genes. Interestingly, we found that genes that are up-, down-, or transiently upregulated in the 24 h state are significantly enriched in regions of high inter-chromosome intermingling in the A compartment (Figure 5C). Moreover, consistent with the 3D structures that show that the formation of multiway hubs and inter-chromosomal intermingling is very closely related (Figures 3B and 3D), differentially expressed genes are also significantly enriched in multiway hubs (Figure 5C).

We then used transcriptomic information from 73 differentiation defective knockout (KO) ES cell lines to identify gene sets that are functionally relevant for the exit from naive pluripotency.^42^ These gene sets fall into the following two groups, both of which are very enriched in group 3 genes: (1) genes that are deregulated by a KO in both 2i/leukemia inhibitory factor (LIF) and 24 h conditions—termed “constitutive response” genes and (2) genes that are not (or only weakly) deregulated by a KO in 2i/LIF conditions but are significantly deregulated in the 24 h state—termed “24 h-induced response” genes. We also identified an extended group of genes that are closely associated with the naive ES cell state (“naive-associated” genes), which are very enriched in group 2A genes. We found that both groups of pluripotency exit genes were significantly enriched in 24 h multiway hubs (Figure 5D).

Thus, genes that are of potential functional relevance for the exit from naive pluripotency are enriched in multiway hubs/regions of high inter-chromosome intermingling in the A compartment in 24 h state cells.

### There is a global change in transcriptional kinetics and a reorganization of enhancer-promoter interactions in the 24 h transition state

Previous work has shown that a loss of PRC1-mediated gene silencing leads to an increase in the activation rate *k*_*on*_ (or burst frequency) of promoters.^43^ We employed a minimal model of transcriptional bursting described by Kim and Marioni^41^ to study the expression kinetics of individual genes using the scRNA-seq data (Figure 5E). Surprisingly, when looking at all active genes—which are concentrated in the A compartment—we found that there is a significant global increase in the burst frequency *k*_*on*_ in the 24 h state compared with naive ES and primed cells (Figure 5F). However, the rate of transcription *s*, i.e., the amount of RNA made each time a gene gets switched on, decreases (Figure 5F). Thus, the overall levels of transcription of most genes remain similar. We found that genes in groups 1, 3, and 2B, whose H3K27me3 levels either decrease or only increase slightly at 24 h, follow this global trend of increased transcriptional bursting (Figure S7). By contrast, transcriptional bursting does not increase in group 2A genes, whose H3K27me3 levels go up across the time course (Figure S7).

**Figure 6:**
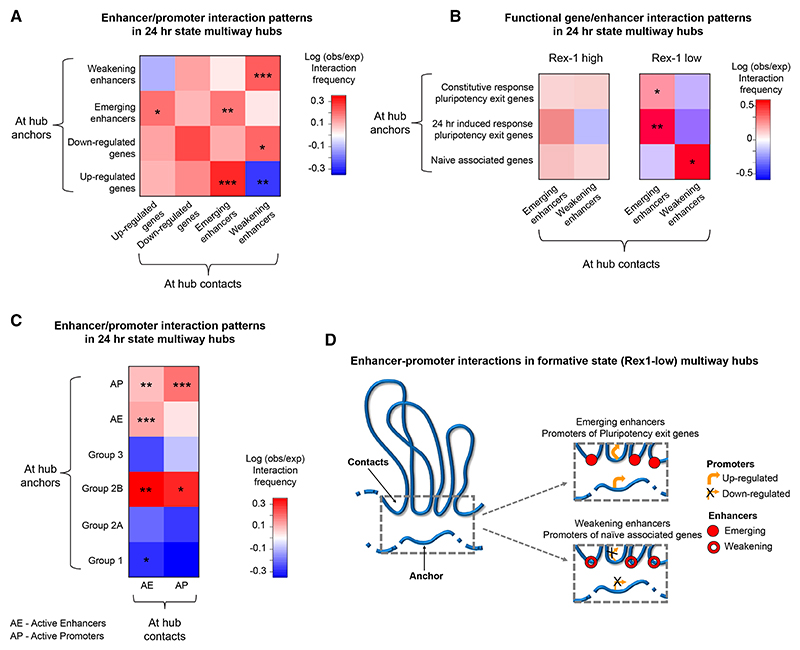
Enhancer-promoter interaction patterns in 24 h state multiway hubs (A–C) Heatmaps showing the observed vs. expected interaction frequency of contacts that form within 24 h state multiway hubs between up-/down-regulated promoters and emerging/weakening enhancers (A), between pluripotency exit/naive-associated promoters and emerging/weakening enhancers (B), or between the different groups of H3K27 methylated genes and active enhancers/promoters (C). In (A)–(C), the colors shown in the heatmaps indicate the log fold change in interaction frequency compared to the randomly expected distribution of the number of hub contacts between regulatory elements. FDR-adjusted empirical p values: * p < 0.05; ** p < 0.01; *** p < 0.001. (D) Schematic illustrating the finding that up-regulated genes contact emerging enhancers, and conversely, down-regulated genes contact weakening enhancers in long-range multiway hubs. Related to Figure S8.

We hypothesized that in the 24 h transition state, the disruption of PRC1/2-mediated heterochromatin and genome structure, together with the increase in transcriptional bursting, might allow new enhancer-promoter interactions to form through a “transcriptional stirring” mechanism.^44^ After assigning the activity of individual enhancers and promoters in naive ES, 24 h state, and primed cells using the ChIP-seq data, we therefore analyzed changes in enhancer-promoter interactions. We found that there is a significant enrichment of interactions between upregulated genes and emerging enhancers and a corresponding depletion in interactions with weakening enhancers (Figure 6A). This was most noticeable for interactions between promoters in hub anchors and enhancers in hub contacts. Furthermore, in 24 h state multiway hubs, we found that promoters of naive-associated genes tend to contact weakening enhancers in cells that have progressed into the Rex1-low formative state (Figure 6B). In contrast, both the constitutive and 24 h-induced response genes required for pluripotency exit tend to contact emerging enhancers in Rex1-low cells (Figure 6B). These trends were much less obvious in the Rex1-high state, where cells are still exiting naive pluripotency.

We found relatively few *inter-chromosomal* interactions in multiway hubs, consistent with the 3D structures, which show that although multiway hubs occur outside the chromosome core, they tend to be buried compared with the most intermingled regions (Figures 3D and S4). Thus, the enrichments of interactions between genes and enhancers in 24 h state multiway hubs are mostly *intra-chromosomal*. Nevertheless, given the close association between multiway hubs and inter-chromosomal intermingling (Figures 3B and 3D), we wondered whether the latter might also lead to a reconfiguration of *inter-chromosomal* enhancer-promoter interactions. However, although both constitutive and 24 h-induced response genes required for pluripotency exit also tend to contact emerging *inter-chromosomal* enhancers in formative state (Rex1-low) cells (Figure S8), the enrichments are much weaker than those observed in *intra-chromosomal* multiway hubs (Figure 6B).

Taken together, these results show that Rex1-low cells may have progressed into the “formative” state because the pluripotency exit genes required for differentiation have established long-range interactions with emerging enhancers in multiway hubs, whereas conversely, naive-associated genes are interacting with weakening enhancers. This switch in the pattern of enhancer-promoter interactions is not properly established in Rex-l high cells, perhaps explaining why Rex1-high (but not Rex1-low) cells can revert to the pluripotent state. We also found that there is a significant enrichment in the interactions between promoters of the group 2B mesodermal/endodermal genes, e.g., *Edar, Fstl3*, and genes in the Hox clusters, with active enhancers and promoters in 24 h state multiway hubs (Figure 6C). This further suggests that structural changes in the 24 h state may set up group 2B enhancer-promoter interactions, priming them for future lineage-specific expression.

### Formation of the formative state structure is controlled by DNMT3a/3b and TET1

**Figure 7:**
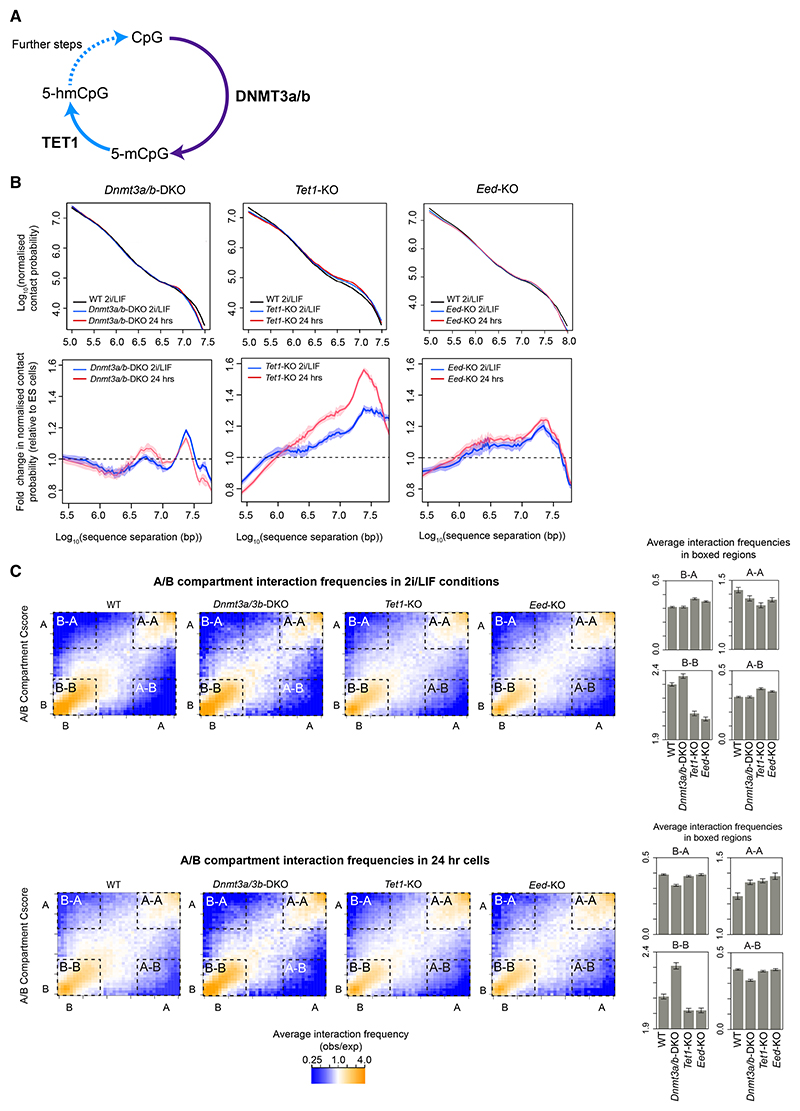
Progression to the formative state genome structure is controlled by DNMT3a/3b and TET1 (A) Scheme showing how DNMT3a/b and TET1 respectively methylate and demethylate CpG sequences to control the levels of DNA methylation at CpG islands. (B) Plots of log_10_ contact probability (upper) and fold change in contact probability relative to that in naive ES cells (lower) against log_10_ intra-chromosomal sequence separation determined using in-nucleus Hi-C experiments carried out on large numbers of cells. Error bands depict the 95% confidence interval. (C) Saddle plots showing the normalized average A/B compartment interaction frequencies between pairs of 25 kb bins, arranged by their A or B compartment Cscores (see STAR Methods for the definition of the Cscore). The bar charts show the average normalized Cscores from within the boxed A/B compartment regions. Bootstrap estimates of the standard deviation were obtained from 200 random samples of 20% of the data. Related to Figure S9.

How might the structural changes we observe in the 24 h state be controlled? In naive ES cells, levels of CpG methylation are low, and during differentiation, they are regulated by the opposing activities of the *de novo* DNA methyltransferases DNMT3a/3b and the 5-methyl-cytosine dioxygenase TET1^45,46^ (Figure 7A). Previous work has suggested that an increase in DNA methylation by DNMT3a/3b could lead to reduced PRC1 recruitment^47–49^ and lower levels of H3K27me3,^39^ and we wondered whether changes in DNA methylation might control the transition to the formative state genome structure. Significantly, in-nucleus Hi-C experiments of *Dnmt3a/3b-*dKO mouse ES cells^50,51^ showed that there was very little change in Hi-C contact probability or A/B compartmentalization 24 h after exit from the naive state (Figures 7B and 7C)—i.e., formation of the 24 h state genome structure requires the activity of *de novo* DNA methyltransferases.

The TET1 enzyme binds to unmethylated CpGs in naive mouse ES cells and co-localizes with most PRC1/2 target genes.^45,46^ Strikingly, we found that genome structure in the *Tet1*-KO^42^ in 2i/LIF conditions resembles that of wild-type cells in the 24 h state—as evidenced by Hi-C contact probability and A/B compartment mixing (Figures 7B and 7C). Allowing cells to differentiate for 24 h caused no further change in A/B compartmentalization, suggesting that in the *Tet1*-KO, the structural changes have already largely occurred in 2i/LIF conditions (Figures 7B and 7C). Importantly, this experiment suggested that the structural changes we observe in the formative state are not due to changes associated with differentiation—because in the *Tet1*-KO, the changes are largely observed in 2i/LIF conditions that maintain cells in a pluripotent state.^52^ Studies of the *Dnmt3a/3b-*dKO and *Tet1*-KO thus suggest that the known increase in DNA methylation^4^ is required for generation of the formative state structure (we also confirmed that levels of DNA methylation increase substantially in multiway hubs at 24 h, data not shown).

Given previous work showing that deletion of *Tet1* can reduce the deposition of both H2AK119ub and H3K27me3,^53^ we next asked whether the changes in H3K27 methylation are linked to the changes in DNA methylation in the formative state. First, we studied A/B compartmentalization in cells where EED, an essential component of PRC2 that catalyzes the formation of H3K27me3, had been knocked out. In naive ES cells, we found similar changes in A/B compartmentalization in the *Eed-KO*, and to a lesser extent, in Hi-C contact probability, to those we had observed in the *Tet1*-KO—suggesting that the structural transformation in the formative state may in part be mediated through changes in H3K27 methylation (Figures 7B and 7C). Second, we used ChIP-qPCR experiments to study changes in H3K27 methylation at selected promoters in all four groups of H3K27 methylated genes (see Figure 4D) in both the *Dnmt3a/3b-*dKO and *Tet1*-KO cells. This showed that the changes in H3K27me3 levels that occur in the naive to formative state transition are not affected by the *Dnmt3a/3b-*dKO, whereas they were completely abrogated in the *Tet1*-KO. At the promoters of groups 1 and 3 genes, H3K27me3 levels were dramatically reduced in naive ES cells in 2i/LIF conditions. By contrast, at the promoters of groups 2A and 2B genes, the levels did not increase in 24 h (formative state) cells (Figure S9).

We conclude that the naive to formative structural transition is regulated by changes in DNA methylation. Although further work is required to determine whether TET1’s control of H3K27 methylation levels is mediated through changes in DNA methylation and/or other mechanisms, the results clearly show that TET1 is required to establish the patterns of H3K27 methylation during early differentiation. The results also show that regulation of the naive to formative structural transition involves more than just changes in H3K27 methylation—a conclusion highlighted by the fact that although the changes in H3K27 methylation occur in the *Dnmt3a/3b-*dKO, the structural changes do not.

## Discussion

Our finding that the formation of multiway hubs in the formative state leads to a reconfiguration of enhancer-promoter interactions as the genome goes through a dramatic transient structural reorganization (see Figure 6D) suggests a hitherto underappreciated role for much longer-range enhancer-promoter interactions. By showing that interactions between emerging enhancers and pluripotency exit genes have become established in cells that have progressed into the formative state (Rex1-low), but not in cells that are less differentiated (Rex1-high), our 3D structures suggest that the formation of unusually long-range enhancer-promoter interactions in multiway hubs could be functionally important.

The potential functional relevance of multiway hub formation, and the long-range enhancer-promoter interactions we observe, is supported by the association of groups of genes whose H3K27me3 levels change in different ways—which are enriched in multiway hubs (Figure 4E)—with alternative developmental lineages (Figure S6A). It is also strongly supported by new studies published while our paper was under review. Notably, highly multiplexed super-resolution FISH imaging around the human *SOX9* and the mouse *Pitx1* loci has identified the formation of multiway hubs and domain compaction, driven by multiple Cohesin bridges, to activate these genes in cranial neural crest cells and the hindlimb, respectively.^54,55^ Moreover, in mouse ES cells, multiway hubs have been shown to decompact after degradation of CTCF and particularly Cohesin.^56^ Our preliminary analysis suggests that CTCF/Cohesin are often associated with the shorter-, but not the longer-range, contacts in multiway hubs (Figure S5C). This argues that Cohesin-mediated loop extrusion could lead to the formation of the shorter-range (~1 Mb) contacts. The large number of active (marked by H3K4me3 and H3K27ac) and repressed (marked by H3K27me3) multiway hubs (Figure 4C) suggests that formation of the longer-range (many Mb) contacts in multiway hubs may result from interactions between genomic regions bound by either transcription factors or Polycomb (PRC1/2) complexes. Finally, we found that in 24 h cells, the promoters of meso/endodermal genes establish contacts with active enhancers and promoters in multiway hubs long before they are expressed, suggesting that formation of multiway hubs in the formative state may also prime genes for activation later in development. The observation of multiway hub formation by complementary methods in different biological contexts suggests that it may prove to be a widely employed mechanism to control enhancer-promoter interactions.

It is not yet clear how the formation of multiway hubs and inter-chromosomal intermingling are mechanistically linked. Initially, we wondered whether the structural changes we observe could result from the altered activity of a particular chromatin regulator that leads to global changes in chromatin dynamics, explaining the previously observed differences in the material properties of 24 h nuclei.^8^ However, our studies thus far suggest that there is no global change in either eu- or hetero-chromatin dynamics during this time course of differentiation (data not shown). We therefore posit that loss of PRC1 binding (which increases transcriptional bursting^43^) leads to increased chromatin stirring,^44^ increased inter-chromosomal intermingling, and the generation of multiway hubs through facilitating Cohesin-mediated loop extrusion. This would be consistent with inter-chromosomal intermingling being mainly observed in the A (euchromatic) compartment (Figure 2B). The increased transcriptional stirring could also enable the formation of multiway hubs by facilitating the establishment of new interactions between regions of the genome having similar chromatin states. In this regard, the similar chromatin state of anchors and contacts in hubs (Figure 4C) is noteworthy.

We find that different multiway hubs are established in different individual cells—i.e., the long-range enhancers that interact with a particular promoter varies from cell to cell. At first sight, this result may seem surprising, but it is consistent with the fact that chromosomal structure (both the local conformation of TADs/loops and large-scale folding), as well as the way different chromosomes pack together, varies enormously from cell to cell.^14^ Given the differences in chromosomal structure from cell to cell, we should not expect enhancer-promoter interactions to be the same in different cells. Rather, we speculate that it is much more likely that genes will be reliably expressed in all cells if a promoter can be activated through the interaction with different compatible enhancers in different cells. Until recently, we have lacked the ability to systematically study genome-wide long-range E-P interactions in single cells, but snHi-C and 3D structure determination now allows this. The 3D genome structures of mouse ES cells in the naive, formative, and primed states of pluripotency show that very rapid changes in chromatin structure and enhancer-promoter interactions (over 1–2 cell cycles) can be linked to altered gene expression, and they raise the intriguing possibility that other changes in either cell fate or cell state could involve a similar structural reorganization.

### Limitations of the study

The resolution of the 3D genome structures does not allow the study of very short-range enhancer-promoter interactions, but our goal here was to study changes in structure and genome-wide enhancer-promoter interactions in single cells, which is difficult using other methods. The results clearly demonstrate that promoters of genes required for pluripotency exit establish unexpectedly long-range interactions with different emerging enhancers in different individual cells, and this correlates with increased transcription of those promoters (Figures 6A and 6B). However, we do not yet have evidence to establish that it is these new enhancer-promoter interactions that lead to the increased transcription, and further investigation to establish causality will require the development of experimental tools to study the association of different proteins with multiway hubs and levels of transcription in the same cell as well as a comprehensive investigation of targeted mutants.

## Star⋆Methods

Detailed methods are provided in the online version of this paper and include the following:

- KEY RESOURCES TABLE
- RESOURCE AVAILABILITY
  ⵔ Lead contact
  ⵔ Materials availability
  ⵔ Data and code availability
- EXPERIMENTAL MODEL AND STUDY PARTICIPANT DETAILS
  ⵔ Cell lines
  ⵔ Cell culture
  ⵔ Cell differentiation
- METHOD DETAILS
  ⵔ Population Hi-C protocol
  ⵔ Single-nucleus Hi-C protocol
  ⵔ ChIP-seq and ChIP-qPCR experiments
  ⵔ Single Cell RNA-sequencing
- 3D STRUCTURE CALCULATION, QUANTIFICATION, AND STATISTICAL ANALYSIS
  ⵔ Hi-C sequence data processing
  ⵔ 3D genome structure calculations
  ⵔ 3D genome visualisation
  ⵔ ChIP-seq data analysis
  ⵔ Defining different groups of H3K27me3 promoters
  ⵔ Definition of enhancer and promoter classes
  ⵔ Single Cell RNA-sequencing analysis
  ⵔ Analysis of the kinetics of gene expression
  ⵔ A/B compartment segregation
  ⵔ Chromosome intermingling scores
  ⵔ Identification of single-cell multiway hubs and analysis of enhancer-promoter contacts
  ⵔ Analysis of inter-chromosomal enhancer-promoter contacts
  ⵔ Location of multiway hubs in the 3D structure

## Star⋆Methods

### Key Resources Table

**Table:** 

REAGENT or RESOURCE	SOURCE	IDENTIFIER
Antibodies		
Rabbit polyclonal anti H3K4me3	Diagenode	Cat#pAb003-050; RRID: AB_2616052
Rabbit polyclonal anti H3K27me3	Millipore	Cat#07-449; RRID: AB_310624
Rabbit polyclonal anti H3K9me3	Active motif	Cat#39161; RRID: AB_2532132
Rabbit polyclonal anti H3K27ac	Active motif	Cat#39135; RRID: AB_2614979
Chemicals, peptides, and recombinant proteins		
16% Formaldehyde, Methanol-free	Pierce	Cat# 28908
Critical commercial assays		
NEXTflex Rapid DNA-seq kit 2.0	Illumina	Cat# NOVA-5188-12
Deposited data		
Raw and processed data for Hi-C, ChIP-seq and single cell RNA-seq	This study	GEO: GSE214264
Original code (version of record – see software and algorithms, below, for the current repositories)	This study	Zenodo DOI: https://doi.org/10.5281/zenodo.10654152
Experimental models: Cell lines		
Haploid Rex1GFPd2-IRES-BSD mouse ES cell line	Martin Leeb	Leeb et al.^26^
Diploid RC9 mouse ES cell line	Martin Leeb	Li et al.^57^
Diploid *Tet1* knockout mouse ES cell line (RC9 background)	Martin Leeb	Lackner et al.^42^
Diploid *Dnmt3a/3b* knockout mouse ES cell line (RC9 background)	Austin Smith	Li et al.^50^
Oligonucleotides		
Hi-C library oligos	Stevens et al.^14^	N/A
ChIP-qPCR oligos	This study	See STAR Methods
Software and algorithms		
NucProcess (Hi-C sequence analysis)	Stevens et al.^14^	https://github.com/tjs23/nuc_processing
NucTools (Hi-C data analysis)	This study	https://github.com/tjs23/nuc_tools
NucDynamics (Single-cell genome structure calculation)	Stevens et al.^14^	https://github.com/TheLaueLab/nuc_dynamics
CscoreTool (AB compartment)	Zheng and Zheng^58^	https://github.com/scoutzxb/CscoreTool
Chromosome intermingling and contact hub analysis	This study	https://github.com/TheLaueLab/formative-state-analysis
Nuc3D (Genome structure visualisation)	Stevens et al.^14^	https://github.com/tjs23/nuc_3d Zenodo DOI: https://doi.org/10.5281/zenodo.7121073
Scater (single-cell RNA-seq)	Bioconductor	https://bioconductor.org/packages/release/bioc/html/scater.html
Seurat v3 (single-cell RNA-seq)	Stuart et al.^59^	https://satijalab.org/seurat
Slingshot (single-cell RNA-seq)	Bioconductor	https://www.bioconductor.org/packages/release/bioc/html/slingshot.html
tradeSeq (single-cell RNA-seq)	Bioconductor	https://www.bioconductor.org/packages/release/bioc/html/tradeSeq.html
PoissonBetaSliceSampleGamma (single-cell RNA-seq)	Kim and Marioni^41^	https://genomebiology.biomedcentral.com/articles/10.1186/gb-2013-14-1-r7#MOESM4
Trim galore (ChIP-seq)	Martin^60^	https://www.bioinformatics.babraham.ac.uk/projects/trim_galore
Deeptools V2.5.058 (ChIP-seq)	Ramirez et al.^61^	https://github.com/deeptools/deepTools
Bowtie2 (ChIP-seq)	Langmead et al.^62^	http://bowtie-bio.sourceforge.net/bowtie2/index.shtml
MACS v2.1 (ChIP-seq)	MACS project: https://github.com/macs3-project/MACS	https://pypi.org/project/MACS2/2.1.1.20160309
Samtools (ChIP-seq)	Li et al.^63^	http://www.htslib.org
PANTHER (GO term analysis)	Mi et al.^64^	http://pantherdb.org
REVIGO (GO term analysis)	Supek et al.^65^	http://revigo.irb.hr
Other		
Protocol for Single-cell Hi-C	Lando et al.^19^	http://www.nature.com/doifinder/10.1038/nprot.2018.017
Protocol to passage haploid mouse ES cells	Leeb et al.^66^	https://doi.org/10.1002/9780470942390.mo140214

## Resource Availability

### Lead contact

Further information and requests for resources and reagents should be directed to and will be fulfilled by the lead contact, Ernest Laue (e.d.laue@bioc.cam.ac.uk).

### Materials availability

No unique reagents were generated in this study.

## Experimental Model and Study Participant Details

### Cell lines

The mouse ES cell lines employed have all been described previously (see key resources table). The haploid mouse embryonic stem (mES) cell line containing the Rex1-GFPd2-IRES-BSD reporter was sorted every 4-6 passages to enrich for haploid cells.^66^

### Cell culture

For routine cell culture, cell lines were maintained in N2B27 media supplemented with 2i inhibitors and mouse leukemia inhibitory factor (mLIF).^52^ 50 % DMEM/F-12 medium (Gibco, 21041025) and 50 % Neurobasal™ Medium (Gibco, 12348017) was supplemented with 1 x N2 to a final concentration of 2.5 μg/ml insulin (provided in-house by the Cambridge Stem Cell Institute), 0.5x B-27™ Supplement (Gibco, 17504044), 1x minimum essential medium non-essential amino acids supplement (Sigma-Aldrich, M7145), 2 mM L-glutamine (Life tech, 25030024), 0.1 mM 2-mercaptoethanol (Life tech, 21985023), 2i inhibitors (1 μM PD0325901, 3 μM CHIR99021) and 10 ng/ml mLIF (provided by the Biochemistry Department, University of Cambridge). Cells were routinely screened for mycoplasma contamination.

### Cell differentiation

For differentiation experiments we prepared samples as previously described.^5^ Cells were plated at a density of 10,000 cells/cm^2^ in N2B27 with 2i and no LIF to avoid delays in the onset of differentiation.^67^ After 24 hrs the two inhibitors were removed and cells were grown in N2B27 only media for a further 24 or 48 hrs to generate Rex1-high/Rex1-low (formative) or primed state cells. To obtain pure Rex1-high and -low fractions, cells at the 24 hr stage were FACS sorted with gates set to collect those with the 25% highest and lowest GFP expression. Näve ES state cells were plated at the same density and grown in N2B27 with 2i and LIF for 48 hrs.

## Method Details

### Population Hi-C protocol

Population Hi-C processing to generate ligated contacts was carried out as follows. Four-five million cells were fixed for 10 min in 14 ml of 2% formaldehyde in growth media at room temperature and then the reaction was quenched by the addition of 1 ml of 2 M glycine. Nuclei were then extracted by incubating cells on ice for 30 min (inverting every 10 min) in 10 mM Tris pH 8.0, 10 mM NaCl, 0.2% NP-40 (IGEPAL CA-630) and protease inhibitor cocktail (Roche). After transferring nuclei to a 1.5 ml Eppendorf tube, the pellet was resuspended in 50 ul 0.5% SDS before incubation for 5 min at 62°C. After incubation, 145 ul of Milli-Q water followed by 25 ul of 10% Triton-X100 were added and the nuclei were incubated again for 15 min at 37°C with gentle agitation. The DNA was digested overnight at 37°C with gentle agitation after adding 25 ml of 10x NEBuffer3 and 5 ul of 25 U/ul MboI (NEB). To biotin end-fill the DNA a 50 ul master mix containing the following was added: 0.3 mM each of dCTP, dGTP, dTTP and biotin-14-dATP (Jena Biosciences) together with 40 U DNA Polymerase I, Large (Klenow) Fragment (NEB), in 1x NEBuffer3. After incubation at 25 °C for 1.5 hr with gentle agitation the nuclei were washed twice with 1 ml of ice-cold TBS buffer (10 mM Tris pH 8.0, 100 mM NaCl). The nuclei pellet containing the biotin labelled DNA ends were resuspended in 500 ul ligation solution containing 0.1% Triton X-100, 50 ug Bovine Serum Albumin, and 2000 U T4 DNA Ligase (NEB) in 1x T4 DNA Ligase buffer (NEB). The sample was incubated overnight at 16 °C with gentle agitation, and the next day the nuclei were pelleted and washed with 1 ml of 1x PBS.

All the remaining steps of the protocol, reversing the formaldehyde cross-links, DNA purification and fragmentation, library preparation and Illumina sequencing were carried out as previously described.^14^ Details of the population Hi-C datasets obtained in this study can be found in Table S1.

### Single-nucleus Hi-C protocol

Single-nucleus Hi-C processing and library preparation were carried out as previously described^14,19^ using the haploid mouse ES line containing the Rex1-destabilised GFP reporter. Libraries were sequenced on an Illumina HiSeq platform with 2 x 125 bp paired end reads at the CRUK Cambridge Institute Genomics Core Facility (Cambridge, UK). Details of the single-nucleus Hi-C samples obtained in this study can be found in Table S2.

### ChIP-seq and ChIP-qPCR experiments

Standard protocols were used to prepare Chromatin immunoprecipitation (ChIP) samples. Briefly, fixation was carried out using 1% formaldehyde for 10 minutes at room temperature and quenched with 150 mM glycine. DNA was fragmented in the presence of 1.0% SDS using a Bioruptor sonication instrument (Diagenode) producing a size range of 200 to 300 bp. Antibodies used for ChIP are listed in the key resources table and the primers used for qPCR are listed in the table below. For quantitative ChIP experiments carried out to study post-translationally modified histones, *Drosophila* spike in chromatin was added to the samples prior to the addition of the antibody. ChIP-seq libraries were prepared using the NEXTflex Rapid DNA-seq kit (Illumina) and sequenced on a Novaseq SP PE50 platform with 2 x 50 bp paired end reads at the CRUK Cambridge Institute Genomics Core facility (Cambridge, UK).

Primer sequences used for the ChIP-qPCR experiments

**Table:** 

Locus	Distance to TSS (kb)	Forward	Reverse
*AVPR1b*	-2.543	AGTGAATGCTGTTGCCGATA	CAGTACTGGGTTTCGGATCG
	-0.827	CCTGCCGTGTAGACTTTCTC	AACTTCTAGCCTCTCTGGGG
	-0.102	GAGCTAGCTTCTCGCTTCTC	GTATTGGAAACCCCTGCCAA
	1.415	GTCATTGTGCTGGCCTACAT	GTGACCCTATTTTCTGTCCCC
	2.255	ATATCATTTGCATGGCCCCT	ATCCTCGGGTTCATGTAGCA
			
*CDH3*	-2.038	ACCCGAGTCTAAATCCCACA	TTGCTCTCCAGAGAATCCCA
	-0.192	ACCTTTAGGGCTTCGAGAGT	CTCAGGTAGCTGCTGATTGG
	0.417	CACACGTGCTTCGCTTTACT	CACAGAAGAGCTGGACGTTAG
	1.151	TGGAGGGTCATTTCGGTACT	GATTCCCTTCCCACGTTCTC
	2.933	GCAGAATAAGGCCTTGGGTT	TGGGAAGTGAGACCTGCATA
			
*FGF5*	-2.556	TAACACCAGCAGGGGACTTA	GGAAACTCTCTGTTGGAGGC
	-1.308	AGTGCTGTCCTGTGAGAGAT	TCAACACAGGAAATCGCCAA
	-0.478	AACAATTCAGGGTGGCTCTC	AACCACTTTCCGAAGGGAAC
	-0.08	TTCTGAGGGCGAGACACCTC	TGTACTCACGGCCGGTACG
	1.432	TAGGCTGAGACCCTGACAAT	AGTGCGAGTGATTAACGTGG
	2.06	ATTCAAGCACAGGTGGGATG	GAACGTCAAGAGAAGGGGTC
			
LAMC3	-3.303	AGGGTAGACCCCAGAATAGC	GGATTGGTGGGACAGCTAAG
	-1.546	TACCCTGACCATGTGGAACT	CCCAATAAGAGGGATGGTGC
	0.431	CCACGGGTAAGGAGTCAGAA	GACCGGATTACAAGTTCGGG
	1.238	TACATACATCCTCAGCACGC	GCTTTTCACTGGGGCATAGT
			
*SLC39a14*	-2.247	TGGTGACCATCACTGTTGTG	GGGCACCCAGAGTTGATAAG
	-1.288	CAGGTAGTTCAGGCATGGTC	AACCGGTTCTGGTAATGCTC
	-0.427	AAACCTAAGGCTTGACCTCG	CCTTCCTAGCCCCTTTTGTG
	0.682	CTCTGGTGTCTGGATCTTGC	TAAAGCTGAACCCACAACCC
	1.524	GGGTCCTCTTTGTGTGACTG	GTCTACATGCAAGGCCAGTT
			
*SPRY4*	-4.745	AAGCCTAGAACTGCAACCAC	CAGCTGGTAGGCTATGTTCG
	-2.082	AAGGGCAAGGAGGGTTATCT	GTGTGATCAGAGTGCCTGAG
	-1.029	CTCTTTTCACCGGTTTGGGT	GGCCTTTGTATTCTTGTGGC
	0.918	TCCTTGGCTATGAGGTTCCA	TCGATGTCTGAGGATAGGGG
	1.595	AATGGCTGGGCATCCATATT	GAAAATGGGGAAATCACGCC
	2.954	GCCTGTTAGATATGGCCTGG	CACATGGAACTCCCTCCTTG
			
*NRG3*	-2.08	TCACTAAGGGGAGAGGGTGG	TCAACCTGGTTCAGTCGGTG
	-1.54	CCTGCATCCAATCTCCTCCC	CCCTCCTGTGGGAAGCTTTT
	-0.78	GTGTTCCGTGCTGTGGTAGA	GCTTCTTCCAGGACGCCTAA
	0.59	CCACCGTGAGTCAAACTCCA	TTGGTATCCTCTGCGTGCTG
	2.12	GGTGCTGCTGTTGACACTTG	TGCAGCACCTCTCTTTGTGT
			
*FGF3*	-2.01	TCCATCAGTGACTCCAGGGT	GAGCACCCTCAACTGTCTCC
	0.82	ACTGCGCTACCAAGTACCAC	CTGTCCGGACCTGAACACTC
	1.63	CCACTCCCCATTTGCTGGAT	CAGACGAGCACCAGACTGAG
	1.76	GTAGAGGCCTCATCCTGCAC	TCAAAGACCCCCGGGATAGT
	2.55	CTTCCAACCAGACCTGTCCC	CCCACTTCCACCGCAGTAAT
			
*GJB2*	-2.44	TCCTGTTTCGCAGTGGACTC	CAGAGCTGAGTGGGTTCCAG
	-1.15	GATACTTCGGGGAGCTGGTG	ACACTTACCCCGGAGTCCTT
	-0.67	GTCAGTGTCTTCCCACGGAG	GTCGCCAGTATTCCTTCGCT
	0.96	CAAGCCCATCCCTCTTGGAG	CCAAGCAATGTGGAACACCG
	2.25	TGTACCCCTTGTTGCACGTT	ACTGGTACTGAGCTCCTCGT
			
*SLC13A5*	-1.62	AACAGGGTGGTCTTTGGTGG	GACTGGGAGGCCATTTTGGA
	-0.46	TCCTGTAGTTCCCTGCCTGA	CCAACGCTAGTCCAGCTCAA
	0.15	GGATCGGGGTGAAGAGCAAA	CGTCCCCCTTTTAAGCTCGT
	0.90	ACGACTTTTACCCTCCAGCG	CCCTCTTTTCTAAGGCCCCG
	2.09	CAGGCCTCATTCAGGACGTT	TAGCTGCAGCGCACACTTTA

### Single Cell RNA-sequencing

At each time point (Naïve ES, 24h and 48h) cells were collected using Accutase. The cell suspension was then sorted using a MoFlo cell sorter (Beckman Coulter) to recover one cell per well in a 96-well plate in 2 μl of cell lysis buffer (0.2% v/v Triton-X100 and 2U/uL of RNase inhibitor). Library preparation was carried out using the Smart-seq2 protocol,^68^ and the libraries were sequenced at the CRUK Cambridge Institute Genomics Core facility (Cambridge, UK) on an Illumina HiSeq4000 sequencer.

## 3D Structure Calculation, Quantification, and Statistical Analysis

### Hi-C sequence data processing

Both single-nucleus and population Hi-C sequencing libraries were processed using our software package “NucProcess”, which is available at: https://github.com/tjs23/nuc_processing/tree/release_1.3

Briefly, this software takes paired FASTQ sequence read files, a reference genome sequence (in this case Mouse assembly GRCm38) and knowledge of experimental parameters (restriction enzyme type and the range of size of DNA fragments sequenced in the library) to create processed, single-cell or population Hi-C contact data.^14,19^ Given that read pairs are not necessarily close in sequence, the two FASTQ files are mapped separately to the reference genome and aberrant, redundant or non-useful pairs (e.g. religations) are discarded. Reads mapping to multiple sequences were kept for single-nucleus Hi-C as positional ambiguity can often be resolved and noise contacts excluded using preliminary 3D genome structures; selecting pairs that are likely close in space.

### 3D genome structure calculations

Using our previously described NucDynamics protocol,^14^ 3D genome structures were calculated using simulated annealing of an initially random conformation of a particle-on-a-string representation of the chromosomes, to generate either 100 kb or 25 kb bead resolution structures that were compatible with the experimental distance restraints derived from the Hi-C contacts. Repeat calculations from different random starting positions were used to generate multiple (usually highly similar) models for each nucleus, which were then aligned spatially. The root mean square displacement (RMSD) value in particle radii between models and restraint violation data for each cell is shown in Table S3.

The software written to perform this task and details of how to install, compile and run it (which includes both a command line version and a Jupyter notebook) is available at: https://github.com/tjs23/nuc_dynamics/tree/release_1.3

### 3D genome visualisation

Genome structures and associated data tracks were visualised using the Nuc3D software available at: https://github.com/tjs23/nuc_3d

This software reads 3D genome/chromosome structures in N3D format; a simple x,y,z coordinate format output by NucDynamics, and renders them in various styles, including as ball-and-stick, line segment or tubes. The user can then zoom, rotate, slice and expand the structures etc., and generate images and movies. Additionally, chromosome data tracks, such as those relating to contact hubs, A/B compartments or histone modifications, may be loaded (typically from a BED format file) and superimposed upon the structures, either as chromosome colours or marker symbols. We have also made a Ubuntu (Linux) virtual machine which has the Nuc3D software installed available for download at: https://zenodo.org/record/7121073#.YzSuuy1Q2Do

### ChIP-seq data analysis

Paired-end reads were trimmed with Trim Galore v0.4.0^60^ using default parameters prior to mapping to the mouse genome reference sequence (GRCm38.p6) using Bowtie 2 v2.1.0.^62^ They were then filtered to retain only those reads with a mapping quality >30 using Samtools.^63^ Peaks were called using MACS2 v2.1. Model-based analysis of ChIP-Seq data was carried out using MACS2 with a minimum FDR cut-off of 0.01 (-q 0.01) to find narrowPeaks (except for H3K9me3 and H3K27me3 which have broad peak features). For samples with the *Drosophila* spike-in, reads were mapped to the *Drosophila* genome (release 6/dm6) using Bowtie 2, but with a mapping quality cut-off >10. The *Drosophila* spike-in read count was quantified using the Samtools Flagstat module. Bigwig files were normalised using the corresponding *Drosophila* spike-in read abundance and generated with the –extendReads option in deep-Tools.^61^ ChIP-seq reads abundance profiles for each ChIP-seq peak were calculated from the normalised bigwig files using ‘bwSummary’ in Deeptools.^61^ Only peaks that appeared in both biological replicates were used (with a minimum peak overlap of 30% between replicates). To compare different time points, we generated a union set of ChIP-seq peaks for all time points and then quantified the average reads abundance of the two biological replicates at each time point.

### Defining different groups of H3K27me3 promoters

H3K27me3 ChIP-seq profile clustering at different time points was carried out using agglomerative hierachical clustering with Ward’s linkage.^69^ Specifically, for each H3K27me3 bound gene promoter, ChIP-seq peak intensity differences between the 24 hr state and naïve ES cells, and between primed and 24 hr state cells, was calculated respectively for each ChIP-seq replicate. This set of intensity differences was then scaled by the maximum intensity across all time points for each gene. Euclidean distances between every pair of different promoters based on the normalised H3K27me3 intensity changes were calculated to construct a dissimilarity matrix which e5 Molecular Cell *84*, 1406–1421.e1–e8, April 18, 2024 was used for hierarchical clustering. H3K27me3 gene clusters based on the above were defined using the ‘cutreeHybrid’ function (setting deepSplit = 1, minClusterSize = 100) within the ‘dynamicTreeCut’ package in R.^70^

To understand the functional role of the different H3K27me3 promoter gene groups we analyzed genes from each cluster using the Gene ontology resource^64^ and used the Revigo tool^65^ to generate plots of the results.

### Definition of enhancer and promoter classes

The following list describes how the activity of enhancers and promoters were classified using the ChIP-seq data:

- ***Active enhancers***: H3K27ac and no H3K4me3 or H3K27me3. (Long H3K27ac ChIP-seq peaks were trimmed to 1000 bp around the center for enhancer analysis.)
- ***Active promoters***: Promoter TSS has H3K4me3, H3K27ac and no H3K27me3
- ***Bivalent promoters***: Promoter TSS has H3K4me3 and H3K27me3
- ***Emerging enhancers***: Are absent in naïve ES but present in 48 hr cells, and have at least a 3x increase in H3K27ac ChIP-seq signal when comparing 48 hr and naive ES cells
- ***Weakening enhancers***: Are present in naïve ES but absent in 48 hr cells, and have at least a 3x decrease in H3K27ac ChIP-seq signal when comparing 48hr and naïve ES cells

### Single Cell RNA-sequencing analysis

RNA-seq libraries were aligned to the mouse reference genome (GRCm38/mm10) with the GSNAP aligner (gmap-2014-12-17)^71,72^ using the parameters -n 1 -N 1 for uniquely mapped reads and allowing for novel splice sites. Gene read counts were obtained using HTSeq (v0.6.1) based on gene annotation from Ensemble release 81. For further analysis the mapped scRNA-seq reads were filtered, normalised and analysed using the ‘Seurat v3’ package in R.^59^ Specifically, low quality cells were filtered out based on a requirement for reads from a minimum of 2000 genes and 300,000 reads/cell, with less than 20% of those reads coming from mitochondrial genes. Outlier cells were further filtered using the ‘isOutlier’ function in ‘scater’^73^ (setting nmads=3, type=‘lower’ and log=TRUE). Only genes with detectable expression in more than 10 cells were kept for further analysis. The most variable genes were found using the Variance Stabilizing Transform (VST) implemented in the ‘FindVariableFeatures’ function in Seurat based on a normalised expression matrix. Potential cell cycle effects were evaluated using the ‘CellCycleScoring’ function, using previously described markers,^74,75^ and cell cycle effects were then removed using the ‘ScaleData’ function (setting vars.to.regress =‘S.Score’, ‘G2M.Score’). After removal of cell cycle effects, Principle Component Analysis (PCA) of the log normalised read count for the 2000 most variable genes was carried out using the ‘RunPCA’ function in Seurat.

Pseudo-time analysis was performed using the ‘slingshot’ package in R.^76^ Here PCA was carried out without scaling the genes by their expression variance using cluster labels identified by a Gaussian mixture model. The cells were then ordered according to pseudo-time using the ‘slingPseudotime’ function. Weights assigning cells to lineages were evaluated using ‘SlingCurveWeights’. Genes with significantly variable expression over the time course were identified using the ‘tradeSeq’ package,^77^ through modelling the relationship between gene expression and pseudo-time using a Generalised Additive Model (the ‘fitGAM’ function, after obtaining a *p* < 0.01 using the ‘associationTest’ function in ‘tradeSeq’). Significantly differentially expressed genes were further classified according to their expression level changes into up-, down-, or transiently up-regulated groups through hierarchical clustering using Ward’s linkage.^69^

### Analysis of the kinetics of gene expression

The kinetics of stochastic gene expression were investigated using the Poisson-beta statistical model^41^ and PoissonBetaSliceSampleGamma software available at: https://genomebiology.biomedcentral.com/articles/10.1186/gb-2013-14-1-r7#MOESM4

The software was run on the filtered and normalised RNA-seq reads (see above) with default settings. Genes with zero or extremely low expression were excluded, and those whose kinetics parameters could not be reliably calculated were also removed as described^41^ – in particular, this involved genes with an extremely high *k*_off_ (i.e. >10), whose parameter estimation tends to have poor accuracy.

### A/B compartment segregation

A/B compartments were defined using the Cscore tool^58^ with a bin size of 25 kb. To remove the effect of sequentially adjacent bins having higher numbers of contacts, population Hi-C reads were further normalised by dividing the Hi-C contact scores between two bins by the average Hi-C score between pairs of bins at the same sequence separation within the same chromosome. Cscores for different genomic regions were then quantile normalised and grouped according to their Cscore. For each group of genomic regions with similar Cscores (similar A/B compartment strength) we then calculated the average normalised contact scores between this group and all other Cscore groups with a different A/B compartment strength. The results were plotted in a heatmap showing the enrichment or depletion of contacts between genomic regions with different A/B compartment strength.

### Chromosome intermingling scores

Inter-chromosome intermingling scores defined as ‘the summed length of all genomic regions on other chromosomes divided by the total length of all genomic regions that are in close spatial proximity to a given locus’ were calculated separtately for the A and B compartments using the 3D genome structures, see:

https://github.com/TheLaueLab/formative-state-analysis/

To be defined as within ‘close spatial proximity’ we required the coordinates of the two genomic regions of interest to be well-defined in the 3D structure, i.e. to have an RMSD for the final 10 structural models of <1.5 bead radii. They were also required to be consistently within <2 bead radii of each other in all of the final calculated structures for a particular cell. (Varying this threshold and testing different values gave very similar results.)

To relate the intermingling scores with other features they were partitioned into 5 or 10 different groups by first quantile-normalising the scores from individual cells. For each genomic locus, we then found the median intermingling score (for that particular locus) across all the cells at a specific stage of differentiation. We then used those median scores to partition the scores into equal-sized groups (e.g. the lowest 10%, followed by 10%-to-20% etc.).

### Identification of single-cell multiway hubs and analysis of enhancer-promoter contacts

Multiway hubs were identified by searching through the single cell contact map for each chromosome with a sliding window to identify 40 kb anchor regions where a significantly greater than expected number of long-range contacts with a sequence separation of more than 50 kb are found. Where overlapping hub anchor windows were detected within a cell, we selected the one with the largest number of long-range contacts. To take account of differences in the total number of contacts and contact distribution within each cell, we estimated empirical p-values for the probability of finding an anchor with the observed number of long-range contacts by chance. Specifically, all long-range contacts were randomly permuted 400 times by placing the contact into a different region of the chromosome sequence keeping the sequence separation in the contact the same. We then used the same hub identification algorithm to search across each randomly permuted genome to find the number of long-range contacts to the pseudo anchor. False discovery rate (FDR) adjusted empirical p-values were then calculated for each hub based on the resulting distribution of the number of long-range contacts to the pseudo anchors. Only hubs with FDR adjusted empirical p-values of < 0.05 were kept.

To analyse the contact frequencies of regulatory elements within hubs, the activity of different anchors and contacts was classified based on either the expression changes of genes whose TSS was < 5 kb away, or the histone protein binding profile centered around the end of the contact (see ‘*Definition of enhancer and promoter classes*’, above). Enhancer-promoter (E-P) interactions were called when a Hi-C contact was detected between a promoter and an enhancer if they were no more than 20 kb from the centre of the anchor or 20 kb from the Hi-C contact site. This analysis was carried out based on the following reasoning: first, a lot of evidence suggests that enhancers don’t actually have to be in contact with promoters to function; second, the smallest particle size that we can use when calculating 3D structures (~25 kb) suggests that the single cell Hi-C data determines genome structures to that sort of resolution; third, the analysis of Hi-C experiments carried out on populations of cells is often done by looking at interactions between ~20 kb bins.

The distribution of the expected number of hub contacts between different regulatory elements was estimated by randomly shuffling each anchor and its group of contacts – i.e. anchors were instead paired with sets of contacts from other hubs. When performing this random shuffling, sets of contacts were selected from hubs from within the same compartment, A or B as appropriate. (This is to ensure that the contact enrichment we calculate is not simply due to a global compartmental effect.) The total number of each type of hub contact was then compared to the distribution of values from the randomly shuffled hubs – the *p* values shown are FDR adjusted empirical *p* values obtained from 5000 random permutations. The relevant scripts can be found at: https://github.com/TheLaueLab/formative-state-analysis/

### Analysis of inter-chromosomal enhancer-promoter contacts

To analyse the contact frequencies between promoters from different groups of genes and enhancers in other chromosomes, we used the single cell 3D genome structures to filter out any inter-chromosomal contacts that were not supported by the structure (i.e. not within 3.5 bead radii in the 3D polymer model). Enhancer-promoter (E-P) interactions were called when a Hi-C contact was detected between a promoter and an enhancer if the crosslink was detected no more than 20 kb from the promoter at one end and 20 kb from the enhancer at the other. (Enhancers were classified as described above.) To obtain the distribution in the expected number of inter-chromosomal contacts between different gene promoters and enhancers the filtered single cell inter-chromosomal contacts from either all the Rex1-high or Rex1-low cells were then considered together and the pairing between gene promoters and enhancers was randomly shuffled, keeping the compartment identity of both the same – i.e. genes and enhancers were randomly shuffled within either the A or B compartments, as appropriate. The total number of each type of inter-chromosomal contact was then compared to the distribution of expected values from the randomly shuffled contacts – the *p* values shown are false discovery rate (FDR) adjusted empirical *p* values obtained from 5000 random permutations.

The relevant script can be found at: https://github.com/TheLaueLab/formative-state-analysis/

### Location of multiway hubs in the 3D structure

Beads in the structure were allocated to hubs, A compartment and/or B compartment as appropriate. Inter-chromosomal intermingling was then calculated for each query bead by measuring the spatial density of surrounding beads from all other chromosomes, at the query location. For each query the density was calculated as the sum of one over the inter-bead (center-center) distances. The mean value is then taken over all the structure replicas.

A background ‘all-site’ distribution for a cell/nucleus was calculated by considering all beads and calculating their inter-chromosome densities. For each cell the all density values were scaled by the median (= 1.00) of this background distribution; this allows different cells to be combined with differing global contact densities. The scaled densities for different cells at each developmental stage were then aggregated into a combined set, for each of the A/B/hub classes, prior to making histograms. The relevant script can be found at: https://github.com/tjs23/nuc_tools/blob/master/scripts/plot_hub_anchor_dist_distribs.py

## Supplementary Material

Supplementary material

Table S4

Table S5

Table S6

Vid S1

Vid S2

Vid S3

Vid S4

## Data Availability

The Hi-C, ChIP-seq and single cell RNA-seq datasets reported in this study have been deposited at GEO and are publicly available as of the date of publication. Accession numbers are listed in the key resources table. All the original code has been deposited at Github (with a version of record copied to Zenodo), and is publicly available as of the date of publication. DOIs are listed in the key resources table. Any additional information required to reanalyze the data reported in this paper is available from the lead contact upon request.

## Highlights

- 3D genome structure and H3K27me3 levels are reorganized in formative state pluripotency
- Hundreds of multiway chromatin hubs form in each formative state cell
- Enhancer-promoter interactions are reconfigured in multiway hubs
- The structural reorganization in the formative state is regulated by DNMT3a/b and TET1

## In brief

Lando, Ma, et al. report 3D genome structures of single mouse ES cells as they begin to differentiate and transition through a formative state. Multiway chromatin hubs form in the formative state, providing a place where genes that are going to be turned on establish long-range contacts with emerging enhancers.
