# Supplementary material for: Enhancer-promoter interactions are reconfigured through the formation of long-range multiway hubs as mouse ES cells exit pluripotency
Source: Mol Cell. Author manuscript; Available in PMC 2024 Jun 4. (PMC7616059; doi:10.1016/j.molcel.2024.02.015)
Supplement: Supplementary material [file EMS196544-supplement-Supplementary_material.pdf]

Molecular Cell, Volume *84*

Supplemental information

Enhancer-promoter interactions are reconfigured through the formation of long-range multiway hubs as mouse ES cells exit pluripotency

David Lando, Xiaoyan Ma, Yang Cao, Aleksandra Jartseva, Tim J. Stevens, Wayne Boucher, Nicola Reynolds, Bertille Montibus, Dominic Hall, Andreas Lackner, Ramy Ragheb, Martin Leeb, Brian D. Hendrich, and Ernest D. Laue

Supplementary Information

Supplementary Tables

Table S1. Related to Figure 1. Summary of the population Hi-C data, and statistics for the sequence analysis

Sample	Cell type	Condition	Input reads	Mapped read pairs %	Valid ligation %	Total contacts	Trans %
**Hap_ES_rep1**	Haploid	Naïve ES	177,207,858	57.6	70.2	67,218,209	8.6
**Hap_ES_rep2**	Haploid	Naïve ES	265,388,437	71.6	69.9	110,998,988	9.3
**Hap_24hRexH_rep1**	Haploid	24h_Rex1-High	52,752,503	56.8	80.4	23,733,171	40
**Hap_24hRexH_rep2**	Haploid	24h_Rex1-High	155,278,734	69.5	51.3	38,817,879	23.7
**Hap_24hRexL_rep1**	Haploid	24h_Rex1-Low/formative	65,824,401	57.4	73.9	27,308,454	40.3
**Hap_24hRexL_rep2**	Haploid	24h_Rex1-Low/formative	286,796,047	70.2	50.5	78,538,523	19.3
**Hap_48h_rep1**	Haploid	Primed	172,530,619	50.7	56.1	34,286,170	24.1
**Hap_48h_rep2**	Haploid	Primed	281,747,949	63.4	64.7	68,635,568	26.5
**RC9_ES_rep1**	RC9 diploid	Naïve ES	43,879,391	70.4	69.0	18,331,335	11.4
**RC9_ES_rep2**	RC9 diploid	Naïve ES	43,170,554	71.0	57.2	15,347,877	13.6
**RC9_24h_rep1**	RC9 diploid	24h	23,858,178	59.6	51.4	4,531,234	22.6
**RC9_24h_rep2**	RC9 diploid	24h	33,716,591	69.4	62.1	12,684,662	18.8
**Tet1_ES_rep1**	Tet1KO/RC9	Naïve ES	25,388,389	63.1	83.9	11,704,951	33.1
**Tet1_ES_rep2**	Tet1KO/RC9	Naïve ES	69,662,097	64.2	60.6	20,129,927	35.4
**Tet1_24h_rep1**	Tet1KO/RC9	24h	28,632,985	64.9	83.9	13,954,500	35.6
**Tet1_24h_rep2**	Tet1KO/RC9	24h	81,034,222	65.3	56.4	23,159,908	17.5
**Dnmt3ab_ES_rep1**	Dnmt3abKO/RC9	Naïve ES	20,573,708	63.8	65.3	8,334,738	11.8
**Dnmt3ab_ES_rep2**	Dnmt3abKO/RC9	Naïve ES	40,074,921	67.0	68.2	16,282,008	12.7
**Dnmt3ab_24h_rep1**	Dnmt3abKO/RC9	24h	35,471,889	75.2	85.1	11,477,148	12.5
**Dnmt3ab_24h_rep2**	Dnmt3abKO/RC9	24h	64,594,239	67.9	68.5	27,177,257	14.6
**EED_ES_rep1**	EDDKO/RC9	Naïve ES	27,544,829	70.9	60.5	10,380,766	14.0
**EED_ES_rep2**	EDDKO/RC9	Naïve ES	119,553,485	74.8	63.1	49,954,915	26.5
**EED_24h_rep1**	EDDKO/RC9	24h	20,171,293	69.2	61.8	7,483,070	16.6
**EED_24h_rep2**	EDDKO/RC9	24h	46,893,817	75.8	63.9	19,668,386	25.8

Table S2. Related to Figures 1, 2. Summary of the single nucleus Hi-C data, and statistics for the sequence analysis

Cell	GEO accession	Condition	Input reads	Genome mapped read pairs %	valid ligation %	Final contact pairs	Trans %
**P2E8 (Cell 1)**	GSE80280*	Naïve ES	1,969,076	62.8	89.4	110,042	11.7
**P30E4 (Cell 2)**	GSE80280*	Naïve ES	1,621,648	58.2	92.3	65,636	6.3
**P2J8 (Cell 4)**	GSE80280*	Naïve ES	1,517,614	46.4	89.9	75,740	6.4
**P2I5 (Cell 5)**	GSE80280*	Naïve ES	1,592,161	55.5	89.8	61,855	10.1
**P30E8 (Cell 6)**	GSE80280*	Naïve ES	1,493,430	43.1	92.2	60,334	7.7
**P44F6**	this study	Naïve ES	9,631,683	54.9	85.3	83,849	11.6
**P44F12**	this study	Naïve ES	8,089,512	54.7	84.9	53,089	10.5
**P44H4**	this study	Naïve ES	2,496,195	45.0	82.9	59,919	7.1
**P62E6**	this study	24h_Rex1-High	8,848,740	56.6	85.2	110,545	13.2
**P62E12**	this study	24h_Rex1-High	8,167,306	57.5	79.3	201,451	9.7
**P62F11**	this study	24h_Rex1-High	9,288,453	54.5	85.5	111,412	12.1
**P62G7**	this study	24h_Rex1-High	9,909,863	62.8	50.1	73,969	15.4
**P62G8**	this study	24h_Rex1-High	11,181,667	54.1	77.1	171,538	15.2
**P62H10**	this study	24h_Rex1-High	9,512,686	54.9	81.3	101,843	14.5
**P62H13**	this study	24h_Rex1-High	10,061,265	53.0	68.9	170,648	11.8
**P72E10**	this study	24h_Rex1-High	8,852,510	73.2	38.6	140,169	15.6
**P63E9**	this study	24h_Rex1-Low/formative	13,087,790	55.2	78.3	153,539	13.3
**P63E14**	this study	24h_Rex1-Low/formative	8,999,046	56.3	69.8	153,526	9.71
**P63F8**	this study	24h_Rex1-Low/formative	8,114,855	50.6	83.6	89,588	15.6
**P63G10**	this study	24h_Rex1-Low/formative	8,902,475	43.6	73.1	45,180	15.7
**P63G12**	this study	24h_Rex1-Low/formative	8,593,456	56.4	79.2	138,580	12.1
**P63H7**	this study	24h_Rex1-Low/formative	6,663,739	53.7	79.5	164,059	9.6
**P63H9**	this study	24h_Rex1-Low/formative	6,985,362	56.6	70.0	32,782	15.4
**P63H10**	this study	24h_Rex1-Low/formative	9.830,461	56.4	81.6	96,883	15.2
**P63H14**	this study	24h_Rex1-Low/formative	5,087,395	30.6	81.7	76,832	10.4
**P64E5**	this study	24h_Rex1-Low/formative	9,132,954	57.3	62.9	123,707	14.5
**P64E11**	this study	24h_Rex1-Low/formative	8,764,583	59.9	71.6	170,685	11.3
**P73E8**	this study	24h_Rex1-Low/formative	9,729,185	67.2	47.4	175,663	23.9
**P73F5**	this study	24h_Rex1-Low/formative	9,742,947	48.9	46.9	171,909	18.2
**P73F6**	this study	24h_Rex1-Low/formative	5,638,697	70.9	47.3	63,916	22.1
**P73F8**	this study	24h_Rex1-Low/formative	6,509,055	75.3	31.4	90,002	18.6
**P45F10**	this study	Primed	8,008,518	53.9	77.9	41,921	8.3
**P46D6**	this study	Primed	3,088,951	52.9	75.9	63,035	6.9
**P46D12**	this study	Primed	47,155,006	50.9	76.1	134,838	8.3
**P46G10**	this study	Primed	7,033,068	53.4	77.9	47,890	6.2
**P54E14**	this study	Primed	10,586,024	63.4	31.1	56,418	9.1
**P54F7**	this study	Primed	7,276,700	54.8	77.2	36,819	7.9
**P54G11**	this study	Primed	6,632,760	53.8	78.9	50,729	10.1
**P54G12**	this study	Primed	7,552,400	49.8	75.8	60,671	7.4
**P54G13**	this study	Primed	7,741,535	62.9	20.3	41,198	14.4
**P54H12**	this study	Primed	2,669,034	50.0	81.5	56,298	10.8

* Stevens, T.J., Lando, D., Basu, S., Atkinson, L.P., Cao, Y., et al. (2017), Nature 544, 59-64. (NB – The Cell 1, Cell 2 *etc.* refer to the nomenclature used in that paper.)

Table S3. Related to Figure 2. Summary of the statistics from the 3D genome structure calculation process

			100 kb particles				25 kb particles			
**Cell**	**GEO accession**	**Condition**	**RMSD***	**%V>3**	**%V>4**	**%V>5**	**RMSD***	**%V>3**	**%V>4**	**%V>5**
**P2E8 (Cell 1)**	GSE80280	Naïve ES	0.62	6.12	4.87	4.41	1.34	9.43	6.53	5.40
**P30E4 (Cell 2)**	GSE80280	Naïve ES	0.95	3.06	1.85	1.45	2.10	3.92	2.88	2.24
**P2J8 (Cell 4)**	GSE80280	Naïve ES	1.07	5.66	4.36	3.79	2.25	6.17	5.17	4.56
**P2I5 (Cell 5)**	GSE80280	Naïve ES	1.27	6.18	4.73	4.11	ND	ND	ND	ND
**P30E8 (Cell 6)**	GSE80280	Naïve ES	1.34	4.31	2.77	2.12	ND	ND	ND	ND
**P44F6**	this study	Naïve ES	0.24	2.34	1.67	1.47	0.67	3.41	3.04	2.75
**P44F12**	this study	Naïve ES	0.57	2.39	2.09	1.90	ND	ND	ND	ND
**P44H4**	this study	Naïve ES	0.57	1.26	0.99	0.87	ND	ND	ND	ND
**P62E6**	this study	24h_Rex1-High	0.31	2.87	2.07	1.76	ND	ND	ND	ND
**P62E12**	this study	24h_Rex1-High	0.19	3.17	2.31	2.08	1.26	4.50	3.46	2.95
**P62F11**	this study	24h_Rex1-High	0.13	2.33	1.75	1.55	ND	ND	ND	ND
**P62G7**	this study	24h_Rex1-High	0.25	3.16	2.54	2.27	1.50	7.29	5.67	4.53
**P62G8**	this study	24h_Rex1-High	0.22	3.99	2.89	2.54	1.69	6.38	4.58	3.79
**P62H10**	this study	24h_Rex1-High	0.21	2.85	2.11	1.88	ND	ND	ND	ND
**P62H13**	this study	24h_Rex1-High	0.17	6.55	3.83	2.93	1.36	9.56	7.36	6.12
**P72E10**	this study	24h_Rex1-High	0.28	9.34	6.35	4.91	ND	ND	ND	ND
**P63E9**	this study	24h_Rex1-Low/formative	0.16	3.75	3.03	2.76	1.00	4.99	4.20	3.72
**P63E14**	this study	24h_Rex1-Low/formative	0.31	2.53	1.96	1.81	1.49	3.50	2.87	2.52
**P63F8**	this study	24h_Rex1-Low/formative	0.27	2.60	2.14	2.00	1.48	3.49	3.18	2.87
**P63G10**	this study	24h_Rex1-Low/formative	0.45	3.07	2.58	2.37	ND	ND	ND	ND
**P63G12**	this study	24h_Rex1-Low/formative	0.28	3.00	2.17	1.92	ND	ND	ND	ND
**P63H7**	this study	24h_Rex1-Low/formative	0.22	2.54	1.81	1.63	1.34	3.71	2.92	2.49
**P63H9**	this study	24h_Rex1-Low/formative	0.79	2.79	2.39	2.15	ND	ND	ND	ND
**P63H10**	this study	24h_Rex1-Low/formative	0.26	2.93	2.38	2.17	1.51	3.85	3.45	3.13
**P63H14**	this study	24h_Rex1-Low/formative	0.20	3.64	3.03	2.71	ND	ND	ND	ND
**P64E5**	this study	24h_Rex1-Low/formative	0.17	2.35	1.78	1.61	ND	ND	ND	ND
**P64E11**	this study	24h_Rex1-Low/formative	0.21	2.03	1.53	1.40	1.34	2.92	2.25	1.91
**P73E8**	this study	24h_Rex1-Low/formative	0.21	5.30	3.03	2.38	ND	ND	ND	ND
**P73F5**	this study	24h_Rex1-Low/formative	0.26	3.98	2.40	1.97	ND	ND	ND	ND
**P73F6**	this study	24h_Rex1-Low/formative	0.37	6.25	3.87	2.92	ND	ND	ND	ND
**P73F8**	this study	24h_Rex1-Low/formative	0.29	4.61	2.84	2.24	ND	ND	ND	ND
**P45F10**	this study	Primed	0.76	1.33	1.12	0.97	ND	ND	ND	ND
**P46D6**	this study	Primed	0.51	1.34	1.12	1.03	ND	ND	ND	ND
**P46D12**	this study	Primed	0.44	2.82	1.87	1.50	1.90	12.64	7.61	4.82
**P46G10**	this study	Primed	0.83	1.21	1.08	0.97	ND	ND	ND	ND
**P54E14**	this study	Primed	0.33	2.03	1.59	1.33	ND	ND	ND	ND
**P54F7**	this study	Primed	0.79	4.50	4.23	3.95	ND	ND	ND	ND
**P54G11**	this study	Primed	0.68	1.14	0.98	0.88	ND	ND	ND	ND
**P54G12**	this study	Primed	0.38	1.49	1.21	1.06	ND	ND	ND	ND
**P54G13**	this study	Primed	0.49	2.53	1.99	1.69	ND	ND	ND	ND
**P54H12**	this study	Primed	0.36	1.86	1.39	1.16	ND	ND	ND	ND

* Median model to model root mean square displacement (RMSD) precision, in units of particle radii

[#] The percentage of violated restraints calculated for thresholds of 3.0, 4.0 and 5.0 particle radii, respectively

ND – v alues not determined because 25 kb particle size structures were not calculated for these cells

Supplementary Figures

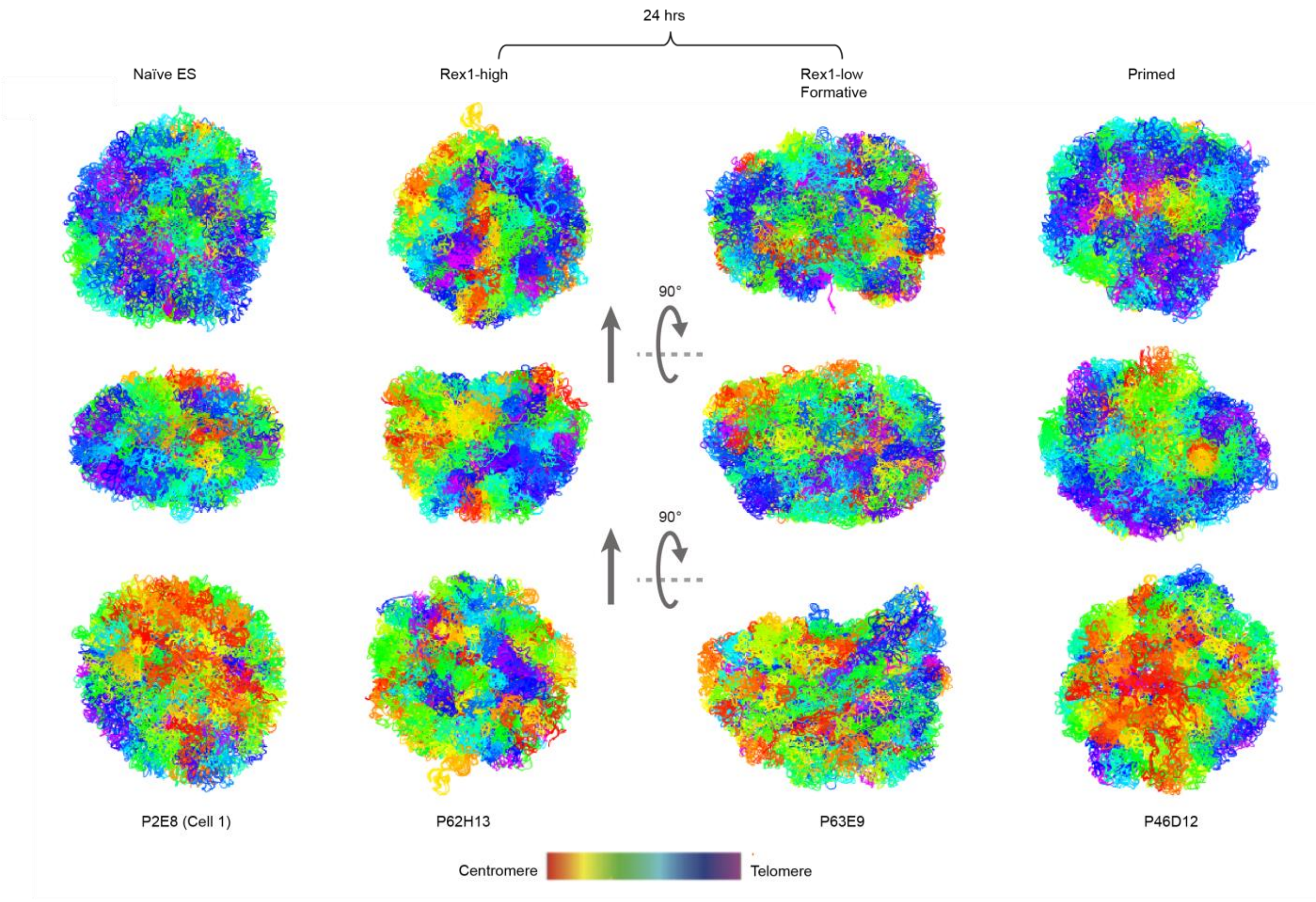

Figure S1. Related to Figure 1. 3D genome structures of representative single G1 phase nuclei, where all the chromosomes are coloured from red to blue (centromere to telomere ends), show a loss of the characteristic Rabl configuration in the 24 hr state.

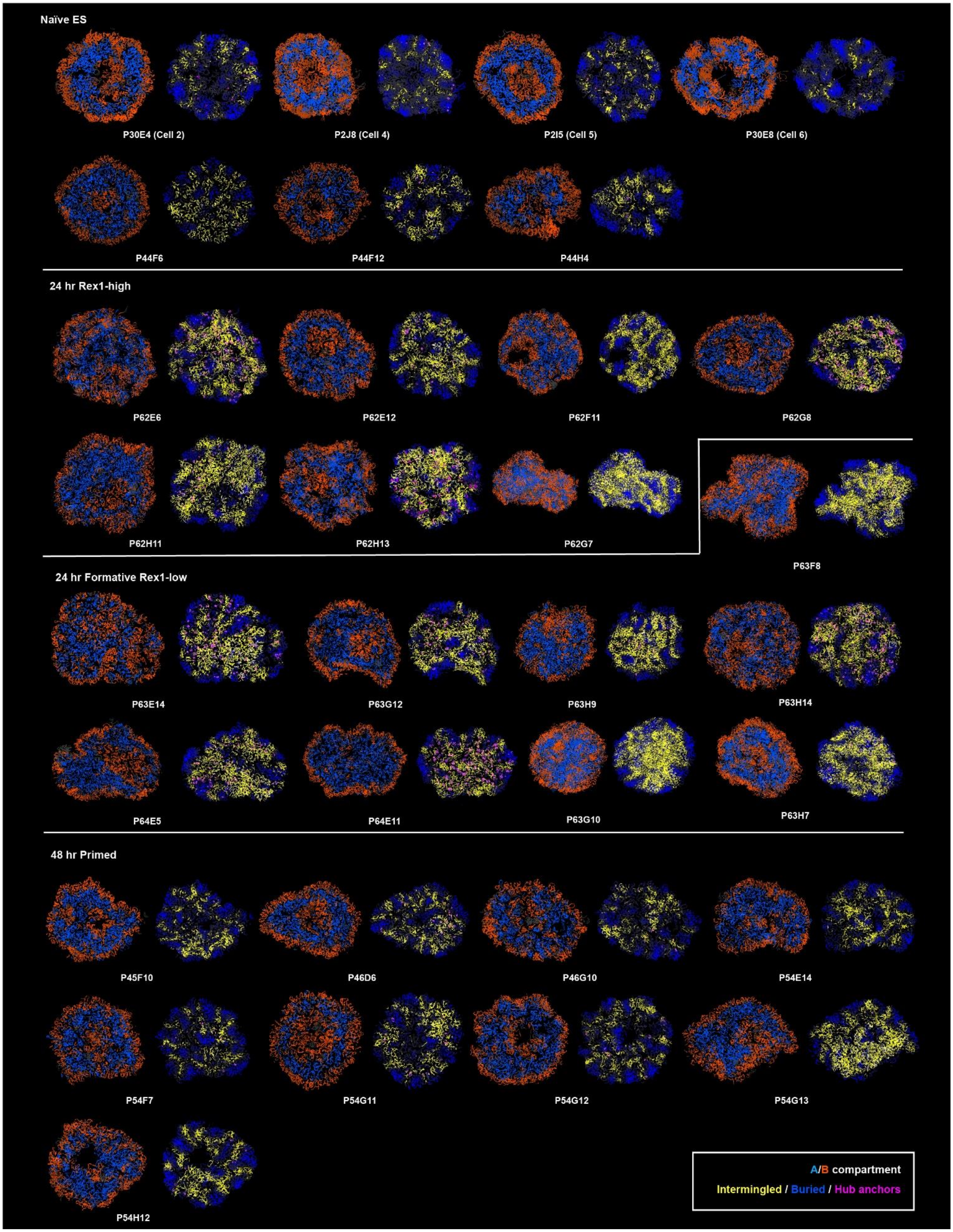
Naïve ES
P30E4 (Cell 2)
P2J8 (Cell 4)
P2I5 (Cell 5)
P30E8 (Cell 6)
P44F6
P44F12
P44H4
24 hr Rex1-high
P62E6
P62E12
P62F11
P62G8
P62H11
P62H13
P62G7
P63F8
24 hr Formative Rex1-low
P63E14
P63G12
P63H9
P63H14
P64E5
P64E11
P63G10
P63H7
48 hr Primed
P45F10
P46D6
P46G10
P54E14
P54F7
P54G11
P54G12
P54G13
P54H12
A/B compartment
Intermingled / Buried / Hub anchors

Figure S2. Related to Figure 2. The structural transition in the formative state involves increased chromosomal intermingling in the A-compartment. Slices through 3D genome structures of further single G1 phase nuclei (see Figure 2A) either coloured blue or red depending on whether a particular region of a chromosome is in the A or B compartment, respectively, or coloured yellow or blue depending on whether that particular region of the chromosome is either intermingled with another chromosome or buried. Regions transitioning between the two are shown in grey.

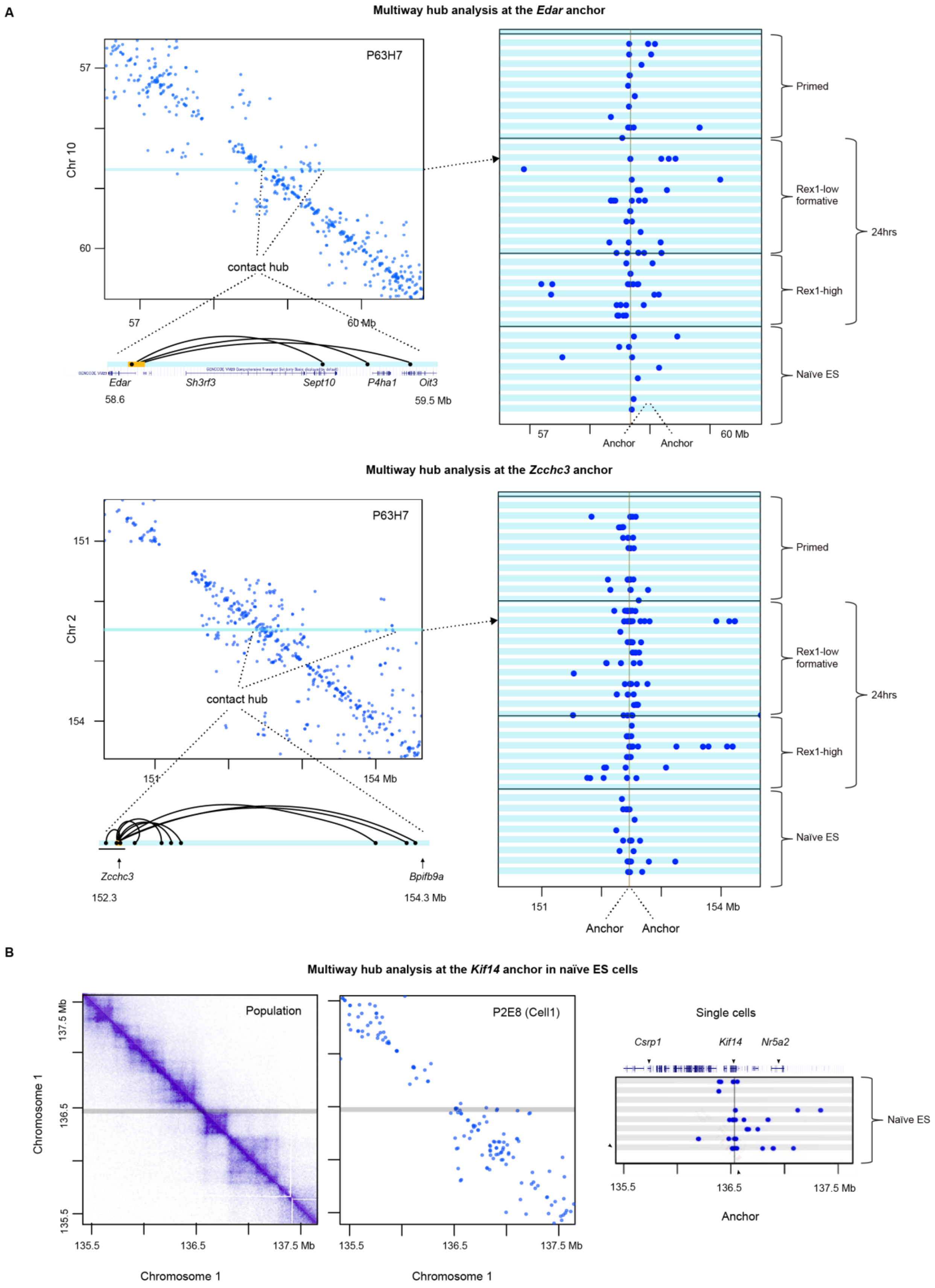
A
Multiway hub analysis at the Edar anchor
P63H7
Chr 10
57
60
57
60 Mb
contact hub
Edar
Sh3rf3
Sept10
P4ha1
Oit3
58.6
59.5 Mb
Primed
Rex1-low
formative
24hrs
Rex1-high
Naïve ES
57
60 Mb
Anchor
Anchor
Multiway hub analysis at the Zcchc3 anchor
P63H7
Chr 2
151
154
151
154 Mb
contact hub
Zcchc3
Bpifb9a
152.3
154.3 Mb
Primed
Rex1-low
formative
24hrs
Rex1-high
Naïve ES
151
154 Mb
Anchor
Anchor
B
Multiway hub analysis at the Kif14 anchor in naïve ES cells
Population
Chromosome 1
137.5 Mb
136.5
135.5
135.5
136.5
137.5 Mb
Chromosome 1
P2E8 (Cell1)
135.5
136.5
137.5 Mb
Chromosome 1
Single cells
Csrp1
Kif14
Nr5a2
Naïve ES
135.5
136.5
137.5 Mb
Anchor

Figure S3. Related to Figure 3. Multiple contacts in 3D genome structures of single cells identify multiway hubs. **A)** Further examples of long range multiway hubs (see Figure 3A) whose 'anchors' are nearby the *Edar* and *Zcchc3* genes. **B)** (Left and Middle) comparison of a small region of the Hi-C contact map from bulk cells with the same region from a single cell. (Right) the contacts made from the anchor region in different single cells. Each horizontal grey box shows the same small section of the contact map (see Middle) from a different individual cell. To allow comparison with published bulk Hi-C data [S1] the example shown here is of a multiway hub that forms in naïve ES cells.

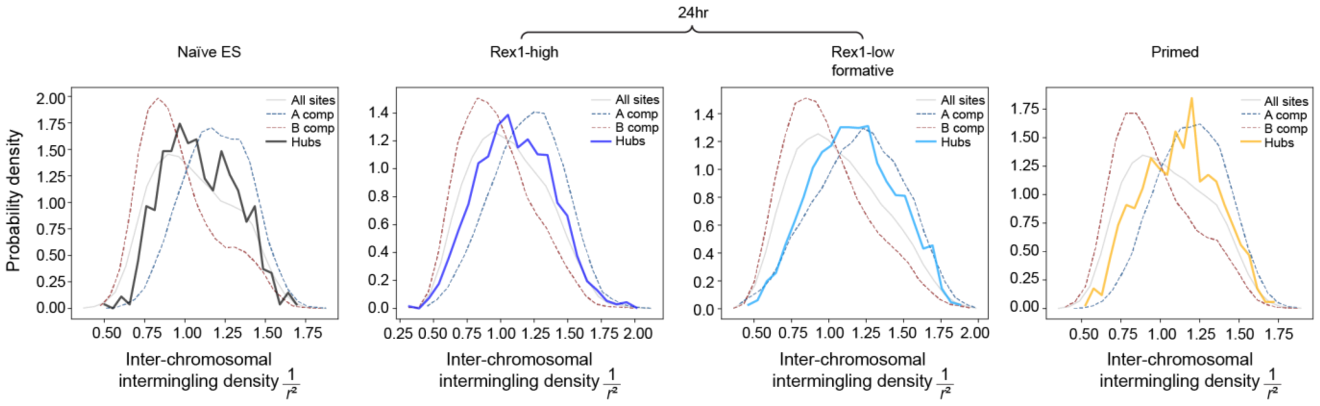

Figure S4. Related to Figure 3. Multiway hubs are located in the regions of chromosomes where the intermingling density is intermediate between that of the A compartment (most intermingled) and B compartment (most buried). Histograms showing the sum of (1/distance^2) from hub anchors (or A or B comparment beads) to beads in all the other chromosomes. The density value increases when more beads from other chromosomes are found at shorter distances, *i.e.* when there is more inter-chromosomal intermingling. (Data from different cells was aggregated after scaling the densities to the median background density value for all sites in each cell.)

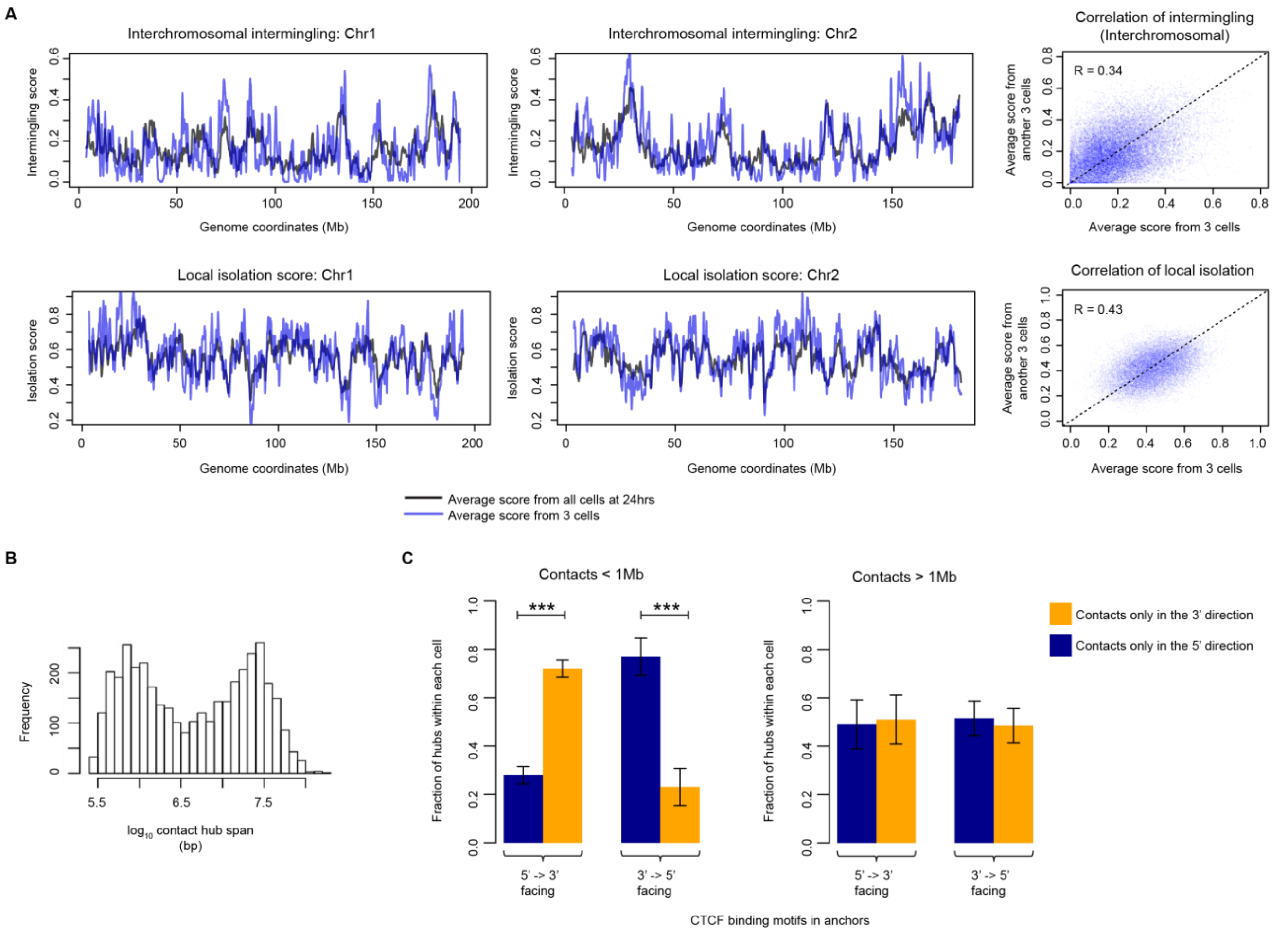

Figure S5. Related to Figure 4. Regions of the genome that are involved in inter-chromosomal intermingling are conserved between cells. **A)** (Left) Plots of the average intermingling scores for two representative chromosomes (1 and 2) from three cells (light blue) overlaid with the average scores for the entire population of 24 hr cell structures (black). (Right) Pearson's correlation coefficient between the average intermingling scores from three cells compared to another three cells. (Similar results were obtained when analysing the separate Rex1-high/low cell structures – data not shown.) **B)** A histogram showing that the span of genome distance for contacts in individual hubs in single cells has a bimodal distribution with average sizes of < 3 or many Mb. (The span is defined as the $\log_{10}$ sequence separation between the most distant positions of intra-chromosomal contacts within a hub.) **C)** Bar charts showing the number of hubs in each cell where all the contacts are found to one side of a CTCF binding motif at the anchor, *i.e.* to either the 3' or the 5' side if the CTCF binding site at the anchor is aligned in either the 5'->3' or the 3'->5' direction. FDR adjusted *p*-values (Fisher's exact test): *** - $p < 0.001$.

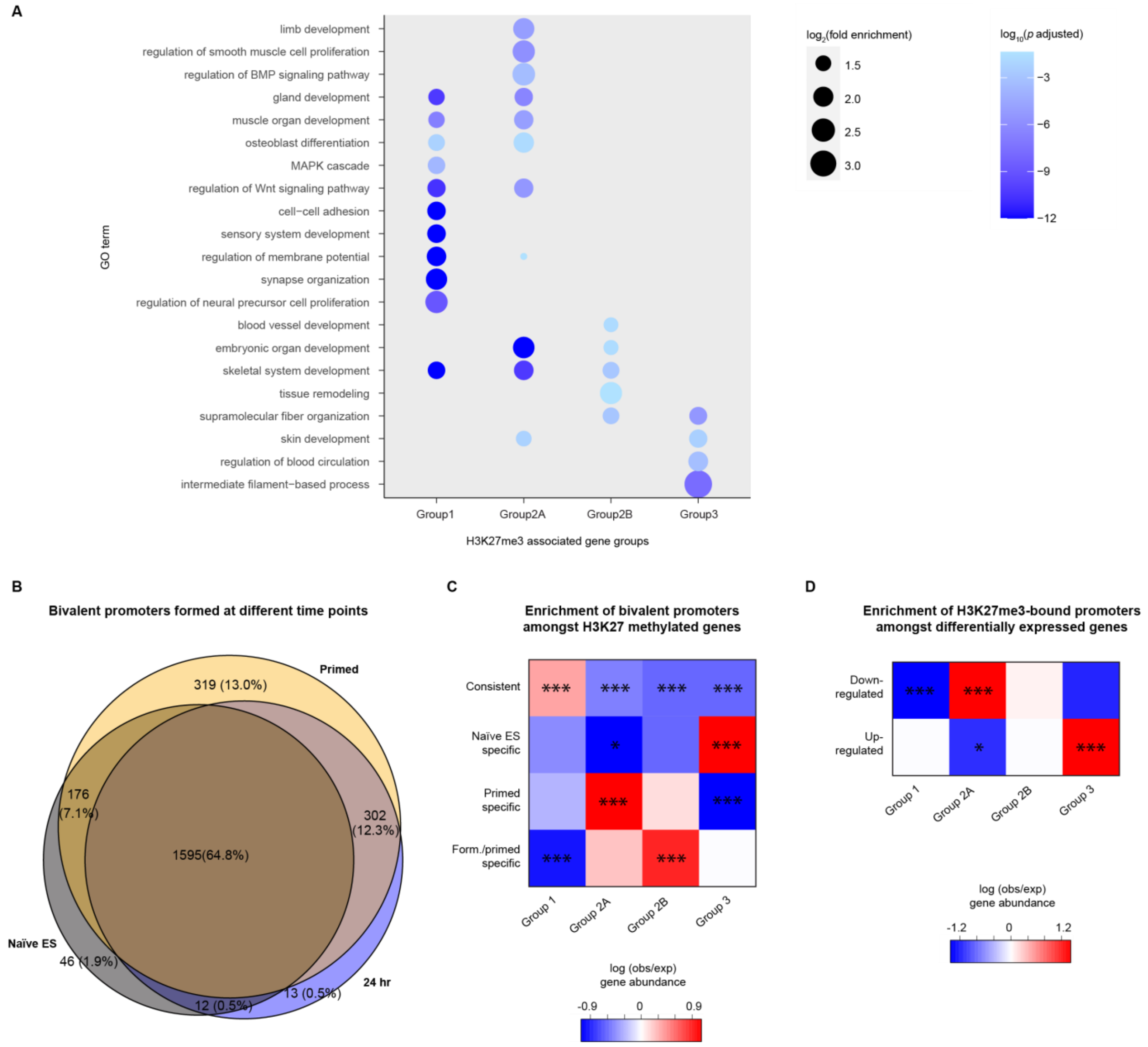

Figure S6. Related to Figure 5. Analysis of the changes in H3K27me3 levels during the time course. **A)** Gene Ontology (GO) enrichment analysis for the different groups of H3K27 methylated genes – the dot-size scales with the Log (fold enrichment), and FDR adjusted *p*-values (Fisher's exact test) are colour coded in different shades of blue. **B)** The numbers of bivalent promoters (bound by H3K4me3 & H3K27me3 containing nucleosomes) formed at different time points in the differentiation pathway. **C)** The enrichment of bivalent promoters formed at different time points amongst the different groups of H3K27 methylated genes. **D)** The enrichment of H3K27me3-bound promoters amongst genes whose expression levels either increase or decrease during the time

course. In C) and D) the colours indicate the observed/expected gene abundance. FDR adjusted *p*-values (Fisher's exact test): * - $p < 0.05$; ** - $p < 0.01$; *** - $p < 0.001$.

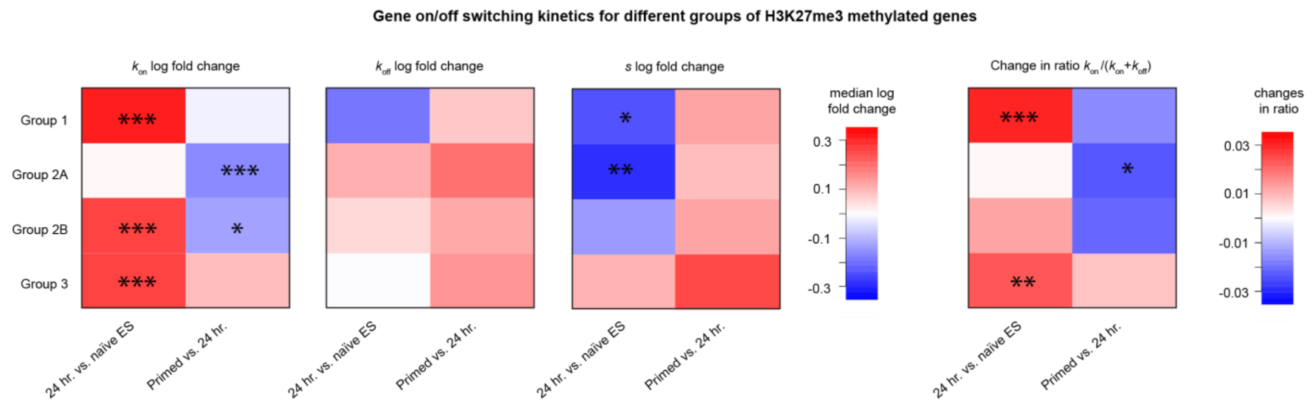

Figure S7. Related to Figure 5. Heatmaps showing the log fold changes (Δ) in the median values of k_{on}, k_{off}, s and the ratio of ($k_{on}/(k_{on}+k_{off})$) for the different groups of genes with H3K27me3-bound promoters. The colours indicate the log fold change in abundance. FDR adjusted p-values (Fisher's exact test): * - $p < 0.05$; ** - $p < 0.01$; *** - $p < 0.001$.

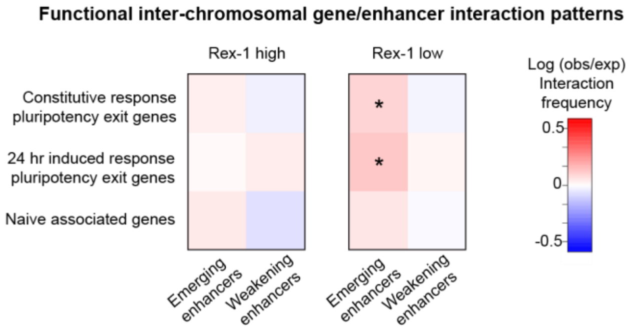

Figure S8. Related to Figure 6. Inter-chromosomal enhancer-promoter interaction patterns in 24 hr state cells. Heatmaps showing the observed vs expected interaction frequency of inter-chromosomal contacts that form within 24 hr state cells between pluripotency exit/naïve associated promoters and emerging/weakening enhancers. The colours shown in the heatmaps indicate the log fold change in interaction frequency compared to the randomly expected distribution of the number of contacts between these regulatory elements. FDR adjusted empirical *p*-values: * - $p < 0.05$.

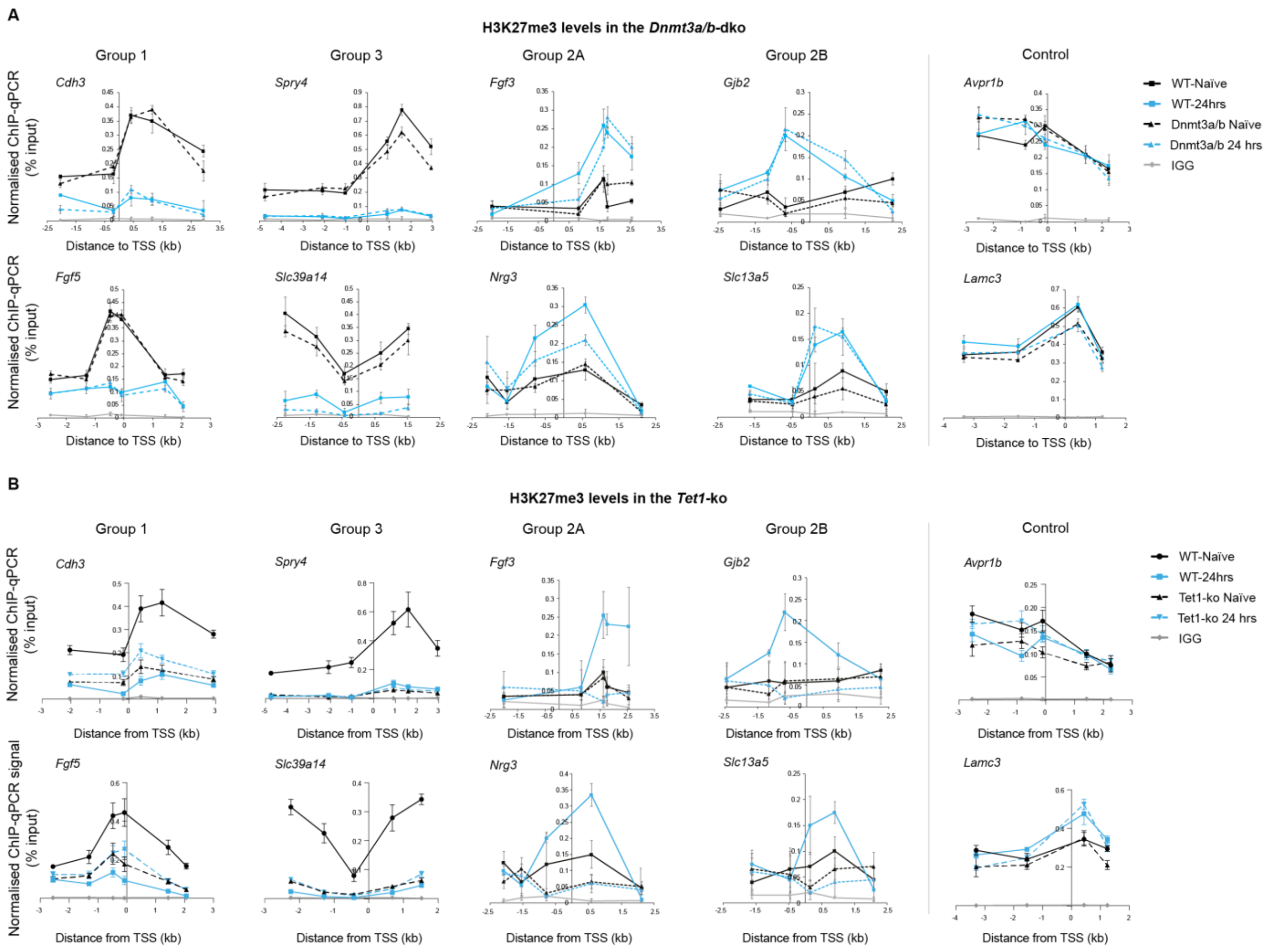

Figure S9. Related to Figure 7. Comparison of H2K27me3 levels in the *Dnmt3a/b*-dko and *Tet1*-ko with wild-type cells [S2, S3] across the promoters of representative Group 1, 2A, 2B and 3 genes as determined using ChIP-qPCR experiments in naïve ES and 24 hr cells. The data are plotted as the mean of three biological replicates and the error bars represent standard error. The controls are genes whose H3K27me3 levels did not change significantly in the ChIP-seq experiments across the time course.

References

S1. Stevens, T.J., Lando, D., Basu, S., Atkinson, L.P., Cao, Y., Lee, S.F., Leeb, M., Wohlfahrt, K.J., Boucher, W., O'Shaughnessy-Kirwan, A., et al. (2017). 3D structures of individual mammalian genomes studied by single-cell Hi-C. Nature 544, 59-64. 10.1038/nature21429.

S2. Lackner, A., Sehlke, R., Garmhausen, M., Giuseppe Stirparo, G., Huth, M., Titz-Teixeira, F., van der Lelij, P., Ramesmayer, J., Thomas, H.F., Ralser, M., et al. (2021). Cooperative genetic networks drive embryonic stem cell transition from naive to formative pluripotency. EMBO J 40, e105776. 10.15252/embj.2020105776.

S3. Li, M., Yu, J.S.L., Tilgner, K., Ong, S.H., Koike-Yusa, H., and Yusa, K. (2018). Genome-wide CRISPR-KO Screen Uncovers mTORC1-Mediated Gsk3 Regulation in Naive Pluripotency Maintenance and Dissolution. Cell reports 24, 489-502. 10.1016/j.celrep.2018.06.027.
